# Rainfall-induced hydroplaning risk over road infrastructure of the continental USA

**DOI:** 10.1371/journal.pone.0272993

**Published:** 2022-08-31

**Authors:** Kaustubh Anil Salvi, Mukesh Kumar

**Affiliations:** 1 Alabama Transportation Institute, University of Alabama; Tuscaloosa, AL, United States of America; 2 Civil, Construction and Environmental Engineering, University of Alabama, Tuscaloosa, AL, United States of America; Al Mansour University College-Baghdad-Iraq, IRAQ

## Abstract

Extreme rainfall causes transient ponding on roads, which increases the risk of vehicle accidents due to hydroplaning (HP), a phenomenon characterized by reduced friction between the pavement surface and the tires of moving vehicles. Before mitigation plans are drawn, it is important to first assess the spatio-temporal patterns of hydroplaning risk (HpR). This study quantifies HpR over the entire continental USA considering the coupled role of precipitation characteristics and pavement properties. Results show the southern United States to be a primary hotspot of HpR. About 22% of road sections experiencing HpR exhibit an increasing trend in the annual occurrence of HP events with time, indicating a riskier future ahead. Alarmingly, road sections that either experience higher HpR or increasing trend in annual occurrences of HP events are the ones with sizeable traffic. These results emphasize the need to prioritize HP-aware road design, traffic management, and signage in regions with high or fast-evolving risks.

## Introduction

Global road safety report by World Health Organization [1] details some highly alarming facts about road safety. For example, road traffic accidents are the leading cause of deaths for children and young adults (5–29 years) and the 8^th^ leading cause of deaths among all age groups, which is more than the combined deaths from diseases such as HIV/AIDS, tuberculosis, and diarrheal disorders. Amendments in road safety laws (e.g., use of seat belt, drink and drive prohibition) along with better driving practices (e.g., effective signal system, driver training) have helped to stabilize the rate of deaths [1], however, the absolute number of deaths is still increasing. Conditions in the United States of America (USA) are no different with more than 39,888 road traffic fatalities [1] and > 6.8 million vehicles crashes [2] in 2016. Among the factors linked to road traffic accidents including the conditions of pavement, weather, vehicle, and traffic, and the alertness, skill, and experience of the driver, weather is often a significant contributor [3]. The role of inclement hydro-meteorological conditions, such as storm surges, intense rainfall and flooding, and frozen precipitation on delays and increased risk of motor vehicle crashes has been widely studied [4–9]. According to the Fatality Analysis Reporting System (FARS) dataset [10], 114,960 fatal crashes leading to 128,917 fatalities occurred during the rainy weather within the USA in the span of 38 years (1980–2017). Road accidents due to rainfall are largely caused by the reduction in visibility of the driver, and incidence of dynamic hydroplaning [11, 12] (HP). HP is a phenomenon, where water ponding over the pavement reduces friction between the pavement surface and the tires of a moving vehicle causing reduced control over the vehicle’s maneuverability thus increasing the risk of accidents. Causes of HP has been the focus of several studies [11–14], wherein an individual or combined roles of controlling factors such as rainfall, pavement, driver, and tire characteristics on HP have been assessed. In fact, the design and engineering of pavement, tires, traffic signs, and rules often already account for these influences so as to reduce the likelihood of HP, and ensure road safety. In spite of this, HP still occurs from time to time, such as when high intensity rainfall results in the development of thick water film on the pavement.

Considering the ongoing and projected changes in rainfall regimes [15], the risk associated with HP is likely to vary in space and time. The specific objectives of this study are to 1) perform a spatio-temporal quantification of rainfall-induced HP risk (HpR) and 2) assess the traffic volume facing elevated threat. To this end, here, a spatially-explicit assessment of rainfall-induced hydroplaning risk (HpR) is performed over the continental USA (CONUS). The study also identifies regions, where high traffic volumes are exposed to these risks. The results are expected to facilitate informed planning for mitigating HP-related risks. HpR in this study is defined as the average annual frequency of high-risk events (HREs). HREs are the instances, when the estimated hydroplaning speed (HPS) or the vehicle speed at which HP will occur for a standard vehicle configuration (explained in section 3.1) is lower than the speed limit (SL). Notably, for these instances, the likelihood of accident is high even when, the driver adheres to the SL. The analysis is carried out using data of meteorological forcings (e.g., North American Land Data Assimilation System (NLDAS) data [16]), pavement properties (e.g., Highway Performance Monitoring System (HPMS) data [17]), and fatal road accidents (e.g., Fatality Analysis Reporting System (FARS) data [18]). Organization of the manuscript is as follows. Section 2 and 3 describes the data and methodology adopted in this study. Results and discussions are presented and discussed in section 4. Section 5 details the summary and conclusions.

### Data (climate, road network, and accident)

Precipitation and air temperature data from the North American Land Data Assimilation System (NLDAS) project [16] are used. These data are available at 0.125° spatial and an hourly temporal resolution, and have been widely used in a range of disciplines such as hydrology [19], public health [20], and agro-meteorology [21]. Since the focus of this study is on hydroplaning induced by intense rainfall, rainfall events are filtered out from the precipitation data which may also include snow events or events with a mix of snow and rain. As noted in the literature [22, 23], methods used for identifying the phase of precipitation as snow or rain based on the temperature are full of uncertainties. To only select rainfall (liquid phase of precipitation) events, a conservative air temperature threshold of 4°C is used, as precipitation is likely to be in the liquid phase at and above these temperatures [23].

The Highway Performance Monitoring System (HPMS) road network data [17] maintained by the Federal Highway Administration is used to obtain information regarding 1) the number of lanes, 2) annual average daily traffic, and 3) speed limit for the road sections. The data have been extensively used in a wide range of transportation applications [24]. Even though, HPMS is an expansive dataset over the CONUS, there are numerous gaps in the data. For example, data regarding the number of lanes and speed limit (data specifically required for the present study) are not available for all the road sections (S1 Fig in S1 File). Here, only the road sections for which information regarding the number of lanes is available, are considered. Imputation is carried out to fill the gaps in speed limits at the considered road sections. To this end, the missing speed limits are substituted by the modal speed limit for the corresponding national highway system (NHS) category for each state. For example, if the NHS category of a road section in a given state for which the speed limit information is missing is ‘2’, the new speed limit for the road section is set equal to the mode of all road sections belonging to NHS category 2 within that particular US state.

Fatality Analysis Reporting System (FARS) [18] dataset provides details of fatal road traffic accidents within the CONUS. The data include information on drivers, vehicles, and accident circumstances, and is widely used [25] for analysis. The data are compiled from various sources such as police reports, medical records, drivers’ licensing registries, state highway departments, and medical examiner. It is available from 1975 onwards and has evolved with time in terms of the details that are collected and reported when the accident occurs. For example, before 2001, the accidents were reported at the county level. From 2001 onwards, pinpoint locations of accidents in terms of latitude and longitudes are also provided. It is to be noted that while other road accidents data sets that account for non-fatal crashes do exist, such as the General Estimates System and Crashworthiness Data provided by National Automotive Sampling System, these data are (a) not available for the entire CONUS, (b) the locations of accidents even at county scale are non-resolvable, and (c) they only present nationally representative samples of police reported traffic crashes [26]. In contrast, FARS dataset is a complete dataset where every accident involving fatality is included.

## Methods

### Hydroplaning speed estimation

Hydroplaning speed (HPS) in mile per hour is the vehicle speed at which hydroplaning starts to manifest. Here, a two-step procedure is adopted to estimate the HPS. First, the ponded water depth thickness above road surface asperities (inch) is estimated using the equation proposed by Gallaway [27].


$$WD=0.00338\times (L^{0.43}\times R^{0.59}\times {MTD}^{0.11}\times S^{-0.42})-MTD$$ (1)

Here, *WD* is the ponded water depth thickness above road surface asperities (inch), *L* is the flow path length (feet), *MTD* is the mean texture depth of the road (inch), *R* is rainfall intensity (inch/hr), and *S* is the pavement slope (feet/feet). The equation was derived under nine standard configurations of pavement surfaces subjected to varied rainfall intensities over a range of cross slopes (from 0.5 to 8%) and MTDs (0.003 inches to 0.164 inches) [28]. Next, with estimated *WD* as an input, a set of equations are used to calculate the HPS.

$$HPS=26.04\times {WD}^{-0.259},for\;WD<0.1\;inch$$ (2A)

HPS=3.09×A,forWD>0.1inch (2B)

where,

$$A=max\left([\frac{10.409}{{WD}^{0.06}}+3.507],[(\frac{28.952}{{WD}^{0.06}}-7.817)\times {MTD}^{0.14}]\right)$$ (3)

Eq 2 is a modified form of the original equation proposed by Gallaway [27]. Eq (2A) was proposed in the study by Huebner [14] for estimating HPS when WD is less than 0.1 inch. Eq (2B) provides a conservative estimate of HPS for a standard smooth ‘American Society for Testing and Materials (ASTM)’ tire for standard vehicle conditions such as zero tire tread depth (smooth tire) with 24 psi inflation pressure and assuming that hydroplaning occurs at 90% spin-down [29]. Hereinafter, Eqs 1, 2A, and 2B will be referred to as Gallaway-Huebner equations.

The equations are widely used [11, 30, 31], and is implemented in many operational models or tools used by state and or federal agencies in US (e.g., Report No. FHWA-RD-75-11 [28] by Texas Transportation Institute) for estimation of hydroplaning speed. Although, actual incidence of HP depends on many other factors including driver’s skill, vehicle’s and tire’s overall health and configuration, and traffic conditions, aforementioned HPS estimates still denote the speed at which the potential of hydroplaning is high. It is to be noted that other methods also exist for evaluating HPS [32]. They also generally simulate a similar inversely-proportional relation between HPS and ponding depth as depicted in Eq 2. But, alternative methods often need additional inputs [13, 33, 34], such as tire properties (e.g. tire pressure, tread design, etc.). Given that historical data regarding make of vehicles, tire conditions, etc., for all road sections and over time is unavailable over the CONUS, application of alternative equations are likely to introduce additional uncertainty. Considering these advantages and focus of the undertaken research problem i.e., to map HpR, Gallaway-Huebner equations are used in the present study.

As per Eqs 1 and 2, the calculation of HPS requires four inputs: rainfall intensity, cross-slope, mean texture depth, and flow path length. Rainfall intensity (R) is obtained from NLDAS data. As spatially-explicit information regarding the magnitude of pavement cross slope (S) for road sections across the CONUS is not available, here, the design criteria guidelines by Federal Highway Administration [35] that prescribes the cross slope on high speed roads to be 1.5–2 percent is referenced. A 2% cross slope is selected as a representative magnitude for the entire CONUS. Notably, the selection of a large cross slope is a conservative choice that is expected to increase the HPS thus reducing the likelihood of HP. Mean texture depth, (MTD) is a property related to pavement macro-texture (unevenness because of road aggregates with vertical amplitudes between 0.004 to 0.8 inches [36]) that plays a major role in establishing wet weather friction on pavement surfaces [11]. It is another input for which spatially-explicit data do not exist. MTD also changes with time because of pavement wear-out due to friction between tires and pavement surface. Guidelines pertaining to MTD provided in the literature are varied. For example, a few studies reported the 1) range of depth of MTD to be from 0.004 to 0.8 inches [36, 37], 2) the average MTD to be 0.03 inches with a minimum of 0.02 inches for any individual test [38], 3) MTD levels in New Zealand, Quebec, and South Australia are between 0.015 to 0.035 inches (0.4 to 0.9 mm) on higher speed roadways [36], 4) minimum MTD requirement by the Minnesota DOT [39] as 0.04 inches (1 mm) for newly constructed Portland cement concrete (PCC) pavements with a longitudinal artificial carpet drag, 5) upper limit of pavement textures (pertaining to the unevenness of the road aggregates) to be 0.04 inches [11]. Information from the aforementioned studies can be summarized into two points viz. 1) MTD can be between 0.004 to 0.8 inches and 2) commonly used value of MTD ranges from 0.03 to 0.04 inches. Here, four MTD values 0.004 inches, 0.04 inches, 0.4 inches, and 0.8 inches for analysis, with MTD = 0.04 inches referred to as the standard configuration are selected. The selection of multiple MTD values is to allow evaluation of the sensitivity of hydroplaning risks to MTD magnitude. Flow path length (*L*) is the distance traveled by water on the pavement surface, and is a surrogate for flow accumulation per unit width at any given location on the pavement. Because of the lack of spatially explicit data of cross slope, longitudinal slope, and macro roughness, the flow path length is assumed to be equal to the road width, which is calculated by multiplying the number of lanes by 12 (1 lane is ~12 feet in width [35]). Notably, in reality, *L* is expected to be usually larger than the road width within a road section because of the presence of non-zero longitudinal slopes [40]. Pavement roughness, which can result in the formation of localized runoff basins, can also increase *L* at specific locations by routing ponded water from larger contribution areas as against when it flows only along a cross-slope path. It is to be noted that for the considered realistic properties of tire and pavement properties, the estimated HPS is greater than 45mph for the road sections within the CONUS. Hence, for the road sections with speed limits less than 45mph, the Gallaway-Huebner equations will not report any HREs. Assuming other conditions to be the same, higher frequency of HREs are expected on road sections with higher speed limits (> 55–60 mph).

### HpR and trend in HREs

HpR is evaluated as the average of annual frequency of HREs i.e., instances when the estimated HPS is lower than the SL. States that are ‘hotspots’ of HpR are identified based on the 1) total of annual mean of HREs over all the road sections within a state, 2) length-weighted average of HREs across all road sections. Changes in HpR with time are quantified by determining the trend of annual count of HREs. It is obtained using the non-parametric Mann-Kendall trend test. Only statistically significant trends (five percent significance level) are considered for analysis.

### HRE-affected traffic volume

To assess the traffic volume affected by HREs, the total number of vehicles passing through a road section during hour(s) when HPS < SL is obtained. Notably, the data of annual average daily traffic (AADT), which is available for each road section within the HPMS road network data [17], exists only from 2011 to 2017. Hourly data of traffic volumes [41] at continental scale is also only available from 2011 onward (till 2018) and that too for selected road sections (e.g., federal highways). By synergistically using the two data sets, here the hourly traffic volume for each road section have been estimated. To this end, a simple downscaling approach is used. First, an hourly traffic volume fraction time series for a given year and state is obtained using Eq 3.


$${Hourly\;Traffic\;Volume\;fraction}_{i}=\frac{{TV}_{i}}{\sum_{i=1}^{NH}{TV}_{i}}$$ (3)

Here *NH* is the number of hours in the year and *TV*_*i*_ is the traffic volume in ‘i^th^’ hour in the same year. The calculation is performed for each year from 2011 to 2018 for which the hourly data is available. Hence, hourly traffic volume fraction (HTVF) for ‘i^th^’ hour represents the fraction of the total hourly traffic volume in one year. Next, a multiyear average of HTVF is obtained. The averaging is expected to smooth the spikes in the traffic volume due to transient forcings. The calculation is done independently for each state (S2 Fig in S1 File). These curves are then used to estimate hourly traffic volume on each road section by multiplying the multiyear average hourly traffic volume fractions with the latest AADT data of 2017. The generated data provides an estimate of hourly traffic volume for each road section of a given state, assuming multiyear average hourly traffic volume fraction time series for the selected roads in a state is experienced on all roads within the state, and in all years. Notably, the goal here is not to report the exact value of vehicles that were exposed to HREs at different road sections but rather highlight the spatial distribution of locations where traffic volume exposed to HRE are large vs. small.

## Results and discussion

### Spatial distribution of HpR within CONUS

**Fig 1:**
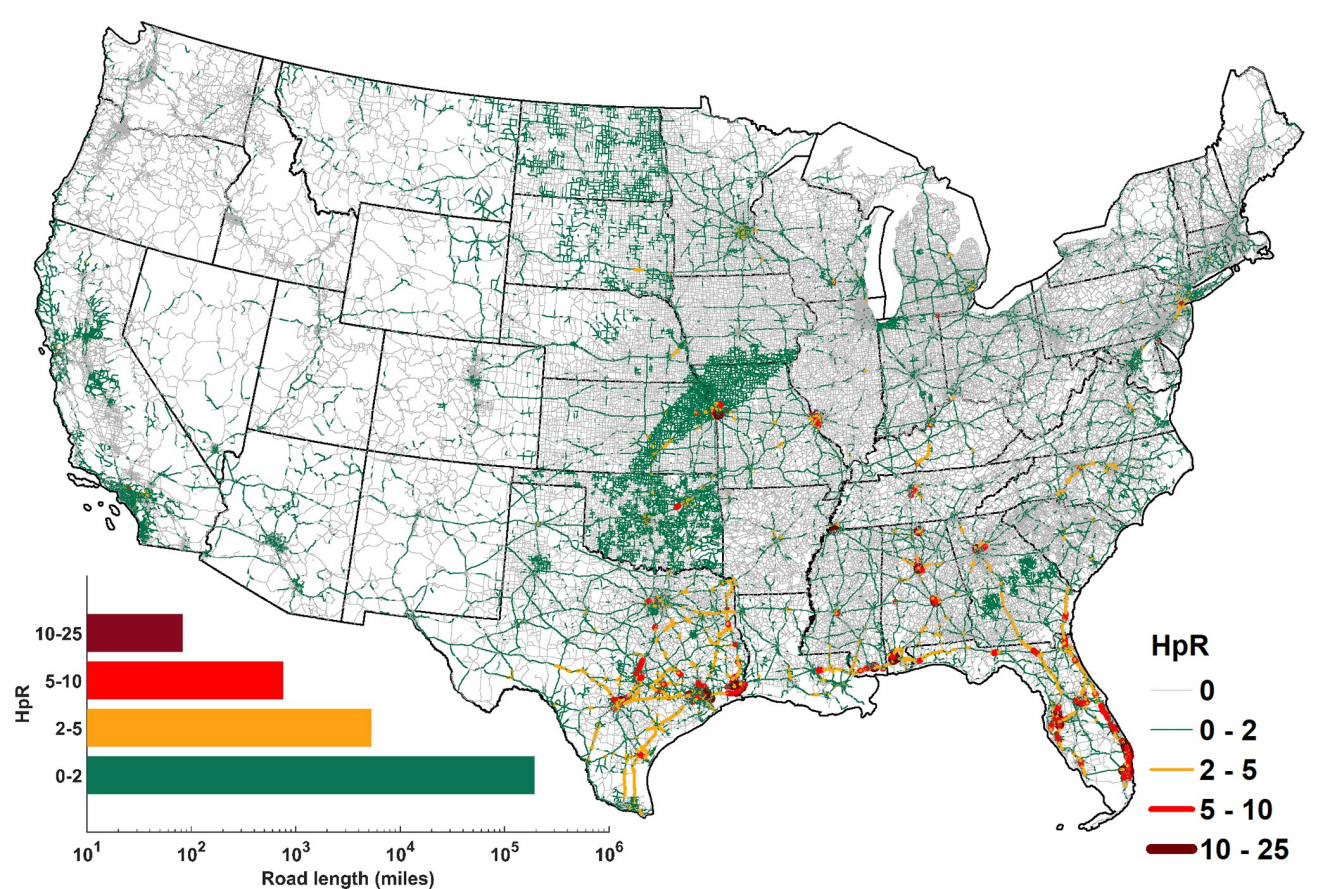
Hydroplaning risk (HpR), quantified as average annual frequency of high risk events (HREs), for road sections within the continental US (CONUS).

HpR is estimated for each road section within the CONUS (section 3.1, 3.2). For a standard pavement mean texture depth (MTD) of 0.04 inch, ~24% of road sections within the CONUS, constituting greater than 200,444 miles of road length (Fig 1), are estimated to experience HpR during 1980–2017. Around 95% of these affected road sections experience <2 HREs per year (Fig 1). While road sections experiencing relatively moderate to high HREs (5–25 per year) are spread across the CONUS, ~87% percent of these road sections (~732 miles) reside within the southern part of CONUS (S1 Table in S1 File). 82 miles of road sections experience a high (10–25) frequency of average HREs per year. Notably, 92% of the 82 miles road sections are situated within the southern states (S3 Fig in S1 File). In terms of the length-weighted average frequency of HREs i.e., the number of annual HREs per unit of road length in a state, and the annual total number of HREs, Texas and Florida take the top two spots (S1 Table in S1 File). Six of the top ten states, viz. Florida, Texas, Alabama, Oklahoma, Mississippi, and Georgia, based on either of the two aforementioned metrics are located in the southern CONUS. Louisiana, another southern state, features in the top ten list of the weighted average frequency of HREs. In summary, southern states not only experience HREs more often but also over longer road lengths.

**Fig 2:**
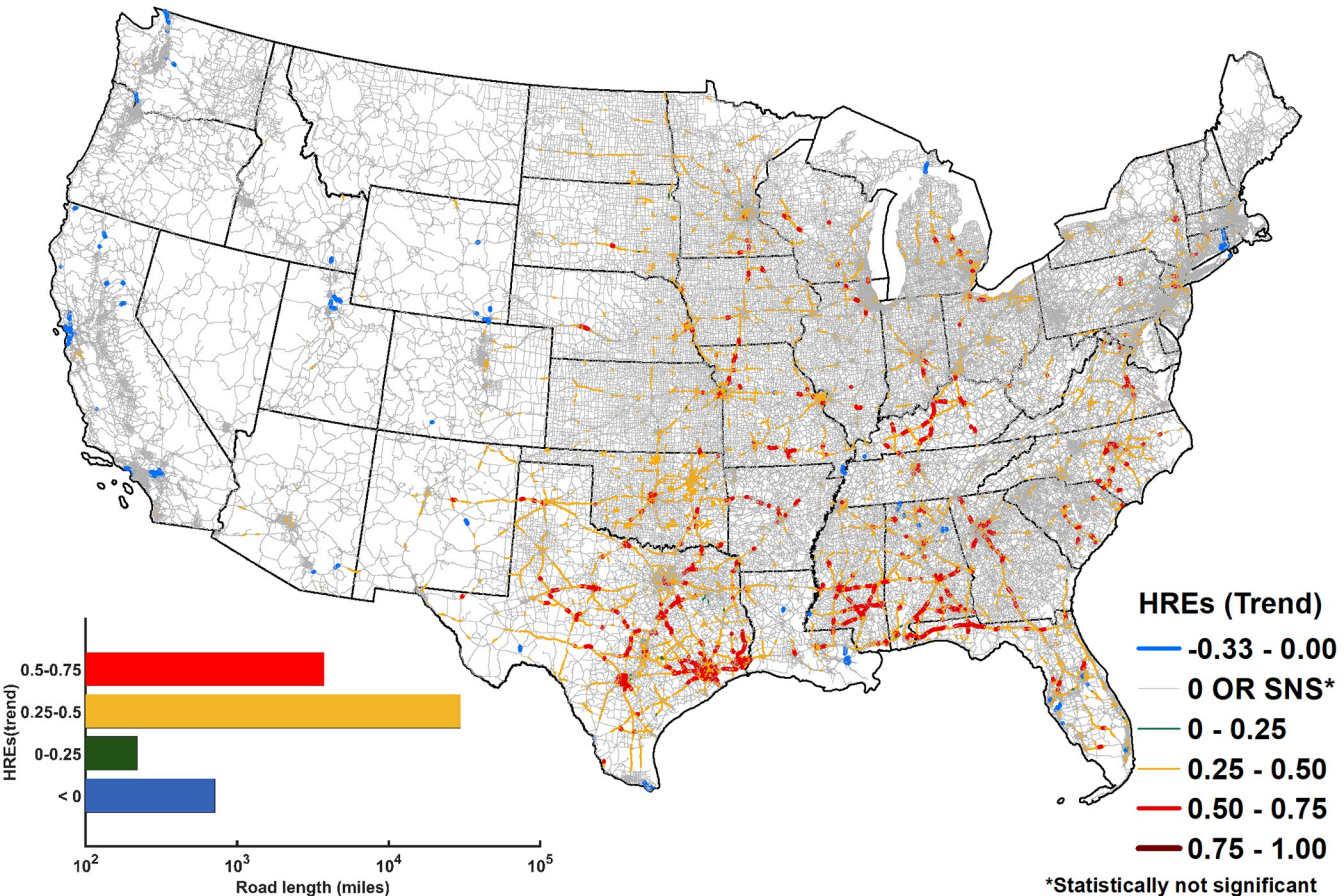
Trends in annual frequency of HREs during 1980–2017. Over ~34,602 miles of road sections (~5.5% of overall road sections within CONUS) experience statistically significant trend (p < 0.05) in annual frequency of HREs. Out of these, 97% of road sections (~33,669 miles) experience moderate to high (0.25–0.75) rate of increase (inset plot). 63% (~21,292 miles) of road sections with statistically significant moderate to high trend reside within the southern United States. 714 miles of road sections (0.12% of roads experiencing HREs) exhibit a negative trend in HREs.

Overall, the road sections experiencing HpR are distributed heterogeneously within a state. HpR is oftentimes more prevalent on wider road sections (i.e., with large number of lanes) that receive intense rainfall. There are a few conspicuous patterns, viz. 1) an unusual triangular region running through Kansas and Missouri (Fig 1) and 2) relatively low HpR (<2) in Pacific Northwest region such as Oregon in spite of receiving heavy rainfall (1800–2500 mm of annual rainfall, S4 Fig in S1 File) is carried out. Further investigation reveals that 1) the triangular HpR pattern is a result of extreme rainfall characteristics prevailing in the region (S5 Fig in S1 File) and 2) low HpR in the Pacific Northwest is driven by narrow pavements (indicating relatively quick removal of ponded water) and lower speed limits (indicating low chances of HPS dropping below speed limit i.e., low HpR). The latter argument is illustrated further through a comparative examination of HpRs in Oregon and Texas (S6 Fig in S1 File), which underscores that HpR is not necessarily high in “wet” regions receiving large annual rainfall, but rather is a function of intense precipitation and pavement characteristics such as speed limit and the number of lanes.

The aforementioned results are obtained while considering a conservative temperature threshold (surface air temperature at height 1.5–2 m from the land surface [23, 42] > 4°C) to qualify the precipitation phase as rain. As the occurrence of rainfall between 0°C and 4°C is not uncommon, to assess the sensitivity of aforementioned results to the choice of temperature threshold, HpRs are also evaluated for a 0°C temperature threshold. Notably, the difference in the temperature thresholds do not affect the results significantly as the overall spatial distribution is very similar in the two scenarios (S7 Fig in S1 File) with the maximum difference in HpRs being restricted to 0.2 per year. Another potential source of uncertainty in the HpR estimate is from the non-availability of spatially-explicit data of MTD. To address this, sensitivity analyses of HpRs over a range of MTDs (0.004, 0.04, 0.4, and 0.8 inch) is performed. Overall, lower (higher) MTD increases (decreases) the extent and frequency of HREs and hence HpR. For example, HREs are observed on as high as 42 percent of the road sections within the CONUS for MTD = 0.004 (S8 Fig in S1 File). Annual frequency of HREs in this case is larger than 35 (at times exceeding 60) for over 5,399 miles of road sections. 94% of the road sections with HREs > 60 reside within the southern states (S9 Fig in S1 File). For higher MTDs such as 0.4 and 0.8 inches (S10 and S11 Figs in S1 File) HREs do not occur. The reason for it is the absence of rainfall intensities that are high enough to produce a water film thickness above MTD of the pavement, leading to HP. Notably, for both smaller MTDs (i.e., MTD = 0.04 inch and MTD = 0.004 inch) considered here, higher frequency of HREs are encountered over southern states, with Texas, Florida, and Alabama featuring in the top five both in terms of number of HREs and length-weighted average frequency of HREs (S1 and S2 Tables in S1 File). Given that MTD = 0.004 inch is quite extreme and less prevalent, the recurrence of southern states on the list points to the robustness of the conclusion that southern states are hotspots of HpR. ‘Flow path length’ is another important factor that affects the occurrence of HREs, but for which the estimates are uncertain. Here, the flow path length is assumed to be equal to road width, given that data of longitudinal slope and macro-topographic roughness are not available at continental scale. An increase in longitudinal slope from 0 to 1% and 2.5% has been reported to increase the flow length by 38% and 158% of road width, respectively [28]. Based on Wqs 1 and 2, this will result in an increase in ponded water depth and hence a decrease in HPS, which in turn will increase the occurrence frequency of HREs. Macro-topographic roughness or undulations in the pavement due to rutting, wear and tear, and design and maintenance imperfections, may also alter the flow length and inundation depth. For example, local depressions on the pavement can act as basins, with rills draining the pavement runoff from the surroundings (S12 Fig in S1 File). This translates to a longer effective flow length (or flow contribution area per unit width at the drainage location) for the pavement runoff w.r.t. the case where runoff flows in a straight line along the cross-slope direction. Longer effective flow length increases the inundation depth, thus decreasing HPS and hence, increasing the occurrence frequency of HREs (based on Eqs 1 and 2). In fact, frequent occurrence of HREs experienced on the pavement with low MTD of 0.004 inch (such low MTDs are far less prevalent) can be matched on a more prevalent MTD of 0.04 inch, if undulations are present. For example, HPS for roads with MTD = 0.004 inches and 8 lanes is matched by pavement with MTD = 0.04 inches and 3 lanes at precipitation intensity of 20 mm/hr (S13 Fig in S1 File), if local undulations increase the effective flow path length to 0.1L^2^ i.e., when the depression drains an effective drainage width of 0.1L and cross-slope length of L. Notably, as both longitudinal slope and macro-topographic roughness manifest locally, and do not exhibit any secular trend over large scales (state or continent), it is likely to not affect the relative distribution of HpRs.

### Evolution of annual frequency of HREs with time

Trends of annual count of HREs during 1980 to 2017 are obtained (Fig 2) using a non-parametric Mann-Kendall trend test. The distribution of trends shows a clear spatial contrast, with the western half of the CONUS (west of Texas) mostly devoid of significant trends (p < 0.05) in the annual frequency of HREs. Over 33,887 miles of road sections (~5.3% of all road section within the CONUS and ~17% by length of the road sections experiencing HREs) show a positive trend of HREs. Most of these road sections (~ 95%) are concentrated in the eastern half of CONUS, although their distribution is quite heterogeneous. More than 219 miles of road sections show a statistically significant positive trend between 0–0.25 HREs/year, 29,908 miles show a positive trend of 0.25–0.5 HREs/year, and 3,760 miles show a positive trend of 0.5–0.75 HREs/year. Out of all the roads within the CONUS with positive trends in annual frequency of HREs, six southern states viz., Florida, Texas, Alabama, Oklahoma, Mississippi, and Georgia, constitute about 51% of these road sections (total length ~ 18,939 miles) (S14 Fig in S1 File). 0.35% of road length experiencing HREs, with a cumulative length of 714 miles, show a negative trend. Notably, a negative trend, while sparse, is prevalent on some roads in a majority of states (S14 Fig in S1 File). California and Louisiana are the top two states in terms of the length of roads exhibiting negative trends in HREs, with 216 miles and 101 miles of road sections respectively. Trend analysis of frequency of rainfall events above a threshold intensity (based on Eqs 1 and 2, this threshold intensity is determined by road characteristics such as its width, slope, MTD, etc.) that would trigger HREs at a selected road sections confirms that the increasing (decreasing) trend in HREs is because of the increase (decrease) in the count of such rainfall events (S15 Fig in S1 File).

Sensitivity of trends of HREs to MTD is evaluated for MTD = 0.004 inch as well (S16 Fig in S1 File). Reduced MTD results in a larger fraction (16% vs. 5.3%) of road sections exhibiting a statistically significant trend in HREs. In all, 149,245 miles of road section show a positive trend (1,689 miles of road sections with trend magnitude between 0–0.25; 119,934 miles with trend magnitude between 0.25–0.5; and 27,621 miles with trend magnitude between 0.5–0.75) and 5,414 miles of road sections show a negative trend of HREs. Southern states contain 63% of road sections with a positive trend (S17 Fig in S1 File). The majority of the road sections with negative trends of HREs (75%) are concentrated within California, Montana, and Wyoming. Interestingly, among the top ten states in terms of lengths of roads showing positive trends in HREs, 6 states (Oklahoma, Texas, Alabama, Missouri, Kansas, Minnesota) also feature in the top ten for MTD = 0.04. This indicates that irrespective of reduction in MTD, spatial pattern of trends do not change significantly.

With reduction in MTD, road sections generally experience intensified trends, i.e., an increase (a decrease) in the magnitude of positive (negative) trend. Overall, the southern states such as Oklahoma (57%), Mississippi (18%), Texas (17%), Alabama (11%), Florida (11%), and North Carolina (9%) experience relatively higher intensification. The numbers within the parentheses represent the percentage of road sections within the state where intensification is observed. A similar situation is encountered for negative trends, where road section mainly within Washington, Utah, California experience intensification.

### Traffic volume in road sections experiencing HREs

**Fig 3:**
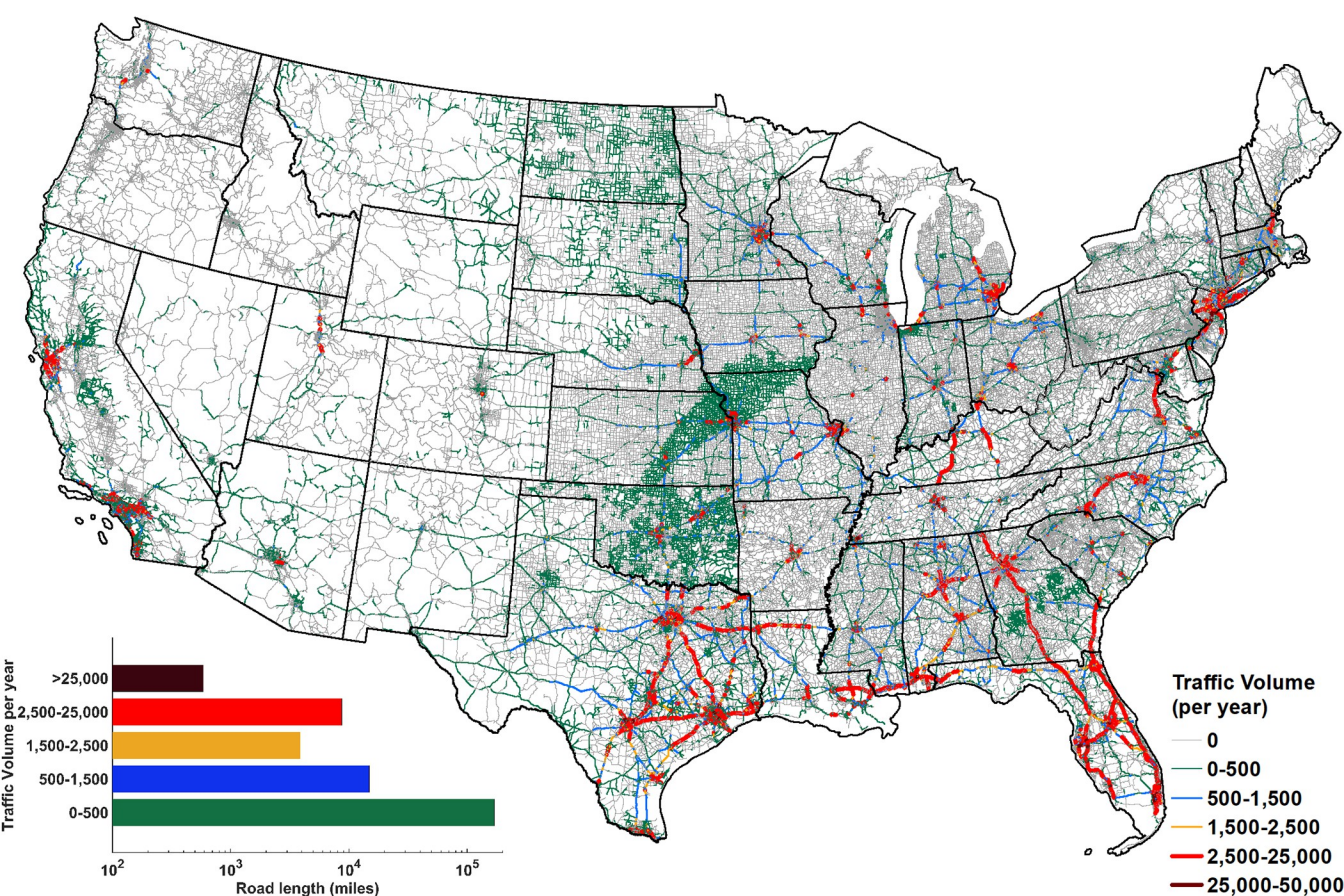
Estimated annual average traffic volume subjected to HpR during 1980–2017. Traffic volume is evaluated over ~198,000 miles or ~98 percent of road sections within CONUS experiencing any HRE. 78% of these road sections constituting 169,981 miles have an annual average traffic volume within 0–500 (inset plot). 58% of the road sections with annual average traffic volume > 2,500 are located within the southern CONUS.

To identify locations where a large number of vehicles are at risk of being directly exposed to HREs or affected by it, first the estimate of hourly traffic for each road section during the occurrences of HREs is obtained. This is achieved by downscaling annual average daily traffic (AADT) data (Methods: HRE-affected traffic volume) to hourly resolution using traffic volume fraction curves of each state (S2 Fig in S1 File). Vehicular traffic on road sections covering over 98 percent of road sections within CONUS experiencing any HRE (~198,000 miles) are estimated (Fig 3). Unsurprisingly, the spatial distribution of intensity of traffic volume exposed to HREs is not the same as that of the HREs (Fig 1), as the traffic volume varies across road sections. A high volume of vehicles getting exposed to HREs at a road section may occur due to high frequency of HREs or very large traffic volumes or both. For example, in California, although relatively low frequency of HREs (0–2 per year) are estimated to occur, because of relatively large traffic volumes, a large number of vehicles may still be affected by HREs.

**Fig 4:**
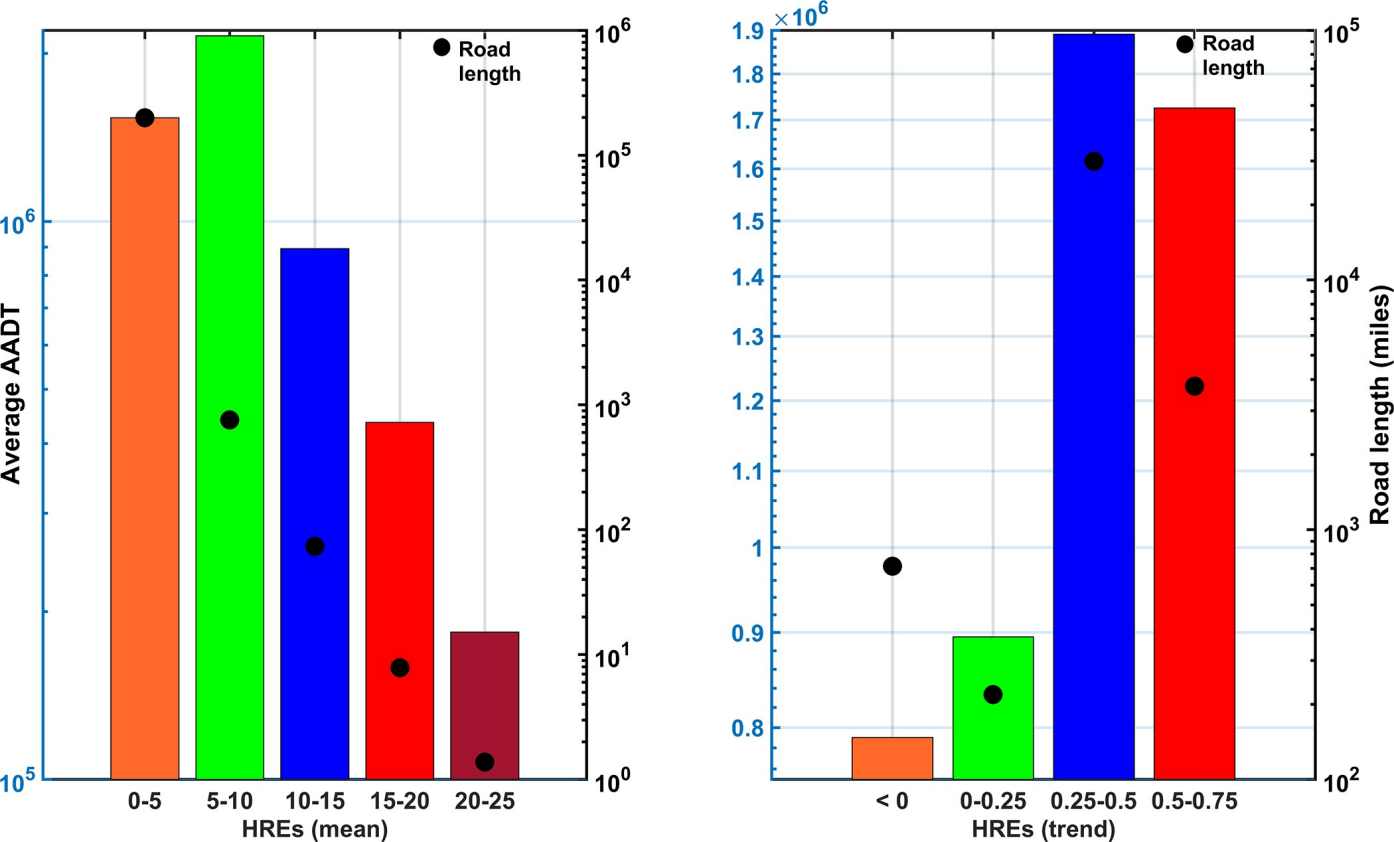
Average AADT over road sections with varied intensity of HpRs and trend in HREs. The evaluation is performed for mean texture depth (MTD) = 0.04 inch. Road sections experiencing HREs > = 5 per year constitute a total length of 840 miles (as compared to 199,604 miles of road sections experiencing less than 5 HREs/year). Notably, the average AADT for these road sections (~3,662,898) is 2.4 times the average AADT (1,533,962) for the road sections experiencing less than 5 HREs/year. This indicates road sections experiencing higher frequency of HREs also support high traffic volumes. High traffic volume also prevails over the road sections experiencing moderate to high (>0.25–0.75) trends of annual HREs.

Analyses over all road sections experiencing HREs within the continental US indicate road sections with annual average HREs between 5 and 10 experience the largest average traffic volumes (Fig 4). Notably, road sections with even higher HREs (10–20) also have appreciable average traffic volumes on them. Another alarming result is that locations experiencing large positive trend (or increase) in HREs (i.e., 0.25–0.75) are where the traffic volumes are high, indicating that if temporal trends of the recent past (last 38 years) continue in the future, road sections with large traffic volumes will be disproportionally affected.

### Nexus between HREs and fatal road accidents

HRE captures instances of elevated accident risks on the road. To assess whether HREs have a direct influence on the number of accidents, however, is challenging. This is due to several reasons, including the fact that road traffic accidents due to hydroplaning may also be influenced by other confounding factors such as driver’s alertness, skill, and experience, and conditions of vehicle and traffic, and inherent uncertainties in the estimation of HP. Furthermore, the data of all accidents (mainly non-fatal), especially those that occur during or due to rain, for different road sections at the continental scale is unavailable. In spite of the aforementioned challenges, to assess if the derived HREs capture situations with elevated accident risk, influence of HRE on fatal road accidents, for which data exists for the CONUS, is evaluated. In this regard, a series of four hypotheses (1_H0–4_H0) are tested to assess if the occurrence of HP and HREs increase the risk of fatal accidents.

1_H0: The proportion/chance of fatal accident for vehicles travelling within the speed limit is higher under conditions with no HP.
2_H0: During rainfall, the proportion/chance of fatal accident for vehicles travelling within the speed limit is higher under conditions with no HP.
3_H0: The proportion/chance of fatal accident for vehicles travelling within the speed limit is higher when HPS > SL.
4_H0: For HP events, the proportion/chance of fatal accident for vehicles travelling within the speed limit is higher when HPS > SL.

The hypotheses testing is carried out for both MTDs i.e., 0.04 and 0.004 inches (refer to Table 1 for details). Testing is carried out using the FARS data during 2001–2017. For this period, the dataset consists of pinpoint latitude and longitude locations of fatal accidents. Accident records where 1) specific locations are not available (missing latitude and longitude of accident locations), 2) speed limits are not known or not reported, and 3) actual vehicle speed is above 151 mph or not known or not reported, are excluded from the analysis. For the selected accidents, the location (latitude and longitude), the day of accident, number of lanes, speed limit, and actual vehicle speed are obtained from FARS dataset. The HPS is estimated using the Gallaway-Huebner equations, where rainfall intensity is obtained from the NLDAS data.

**Table 1:** Details of the four hypothesis tests carried out to assess if the occurrence of hydroplaning and high-risk events increase the risk of fatal accidents.

MTD = 0.04 inch	Total Count	N1	N2	x	y	P1	P2	Z score	P
p1 = The proportion of fatal accidents for vehicles travelling within the speed limit under conditions conducive for HP	218598	1532	217066	1099	131840	0.72	0.61	8.79	< 0.05
p2 = The proportion of fatal accidents for vehicles travelling within the speed limit under conditions NOT conducive for HP									
H0: p1 < = p2									
H1: p1 > p2									
p1 = The proportion of fatal accidents for vehicles travelling within the speed limit during rainfall under conditions conducive for HP	60731	1532	59199	1099	38006	0.72	0.64	6.08	< 0.05
p2 = The proportion of fatal accidents for vehicles travelling within the speed limit during rainfall under conditions NOT conducive for HP									
H0: p1 < = p2									
H1: p1 > p2									
p1 = The proportion of fatal accidents for vehicles travelling within the speed limit when HPS < = SL.	218598	27	218571	21	132918	0.78	0.61	1.81	< 0.05
p2 = The proportion of fatal accidents for vehicles travelling within the speed limit when HPS > SL OR NO HP.									
H0: p1 < = p2									
H1: p1 > p2									
p1 = For HP events, the proportion of fatal accidents for vehicles travelling within the speed limit, when HPS < = SL	1532	27	1505	21	39	0.78	0.03	19.96	< 0.05
p2 = For HP events, the proportion of fatal accidents for vehicles travelling within the speed limit, when HPS > SL									
H0: p1 < = p2									
H1: p1 > p2									
**MTD = 0.004 inch**	Total Count	N1	N2	x	y	P1	P2	Z score	P
p1 = The proportion of fatal accidents for vehicles travelling within the speed limit under conditions conducive for HP	218598	35544	183054	23264	109675	0.65	0.60	19.57	< 0.05
p2 = The proportion of fatal accidents for vehicles travelling within the speed limit under conditions NOT conducive for HP									
H0: p1 < = p2									
H1: p1 > p2									
p1 = The proportion of fatal accidents for vehicles travelling within the speed limit during rainfall under conditions conducive for HP	60731	35544	25187	23264	15841	0.65	0.63	6.49	< 0.05
p2 = The proportion of fatal accidents for vehicles travelling within the speed limit during rainfall under conditions NOT conducive for HP									
H0: p1 < = p2									
H1: p1 > p2									
p1 = The proportion of fatal accidents for vehicles travelling within the speed limit when HPS < = SL.	218598	284	218314	234	132705	0.82	0.61	7.45	< 0.05
p2 = The proportion of fatal accidents for vehicles travelling within the speed limit when HPS > SL OR NO HP.									
H0: p1 < = p2									
H1: p1 > p2									
p1 = For HP events, the proportion of fatal accidents for vehicles travelling within the speed limit, when HPS < = SL	35544	284	35260	214	1145	0.75	0.03	63.11	< 0.05
p2 = For HP events, the proportion of fatal accidents for vehicles travelling within the speed limit, when HPS > SL									
H0: p1 < = p2									
H1: p1 > p2									

Z-score one-tail tests confirm rejection of all four considered hypotheses (Table 1). Rejection of the first null hypothesis (z-score: 8.79, p < 0.05) establishes that the proportion of the fatal accidents encountered by the vehicles moving within SL (hereinafter referred as a regulated condition) is significantly higher when HP is estimated to occur vs. when HP does not occur. Rejection of the second null-hypothesis (z-score: 6.08, p < 0.05) indicates fatality risks are higher under rain events that lead to HP vs. those that do not produce HP. These results confirm that occurrence of HP increases the risk of fatal accidents, and also that HP generating rain events have higher fatality risks than rain events that do not generate HP. Rejection of the third hypothesis (z-score: 1.81, p < 0.05) establishes that under regulated conditions, vehicles face higher risk of fatal accidents when HPS is less than SL. Fourth hypothesis test confirms that among the fatal HP events, the proportion of fatal accidents when vehicle speed exceeds the HPS is more than when HPS is less than SL (z-score: 19.96, p < 0.05). In summary, under regulated conditions, vehicles are likely to face elevated fatal accident risk when HPS drops below the SL, thus further reinforcing that occurrence of HPS < SL or a HRE indeed represents a high risk situation. The four hypotheses are rejected for MTD = 0.004 inch as well (Table 1).

## Conclusions

This is the first study to map the spatio-temporal distribution of rain-induced HP at continental scale, and detect its hotspots i.e., the regions with relatively high HpR. To this end, the study also presents a metric to assess accident risks from HP. Even though the interaction between both pavement and rainfall characteristics determines the distribution of HpR, the metric is demonstrated to capture conditions that incur higher risk to vehicles. Owing to a higher occurrence of intense rainfall, wider road lanes, and higher speed limits, many southern US states are found to have a higher risk of hydroplaning. While road sections experiencing higher frequency of HREs should be prioritized in mitigation planning, such priority should also be accorded to sections experiencing relatively low HREs but with a large traffic volume. Alarmingly, road sections experiencing relatively large positive trend (or increase) in HREs are where the traffic volumes are high, indicating that if the temporal trends of the recent past (last 38 years) continue in the future, road sections with large traffic volumes will be disproportionally affected by increasing HREs, thus exposing more vehicles to it. As the frequency of large precipitation events is projected to become more intense in future [15] with changing climate, traffic risks from HP are likely to get exacerbated.

It is to be acknowledged that the HRE estimates have inherent uncertainties originating from gaps and assumptions in data such as precipitation being uniformly distributed within an NLDAS grid, MTD being identical for all road sections, and pavement surface being perfectly planer with runoff occurring along a straight line. Sensitivity analysis, however, indicates the results regarding the overall regional differences in the occurrence of HREs, and the continental scale distribution of hotspots and temporal trends are robust. The aforementioned results can be used to prioritize pavement-weather forecasts and traffic management and signage in the hotspot regions, and to provide guidelines for road design, especially in regards to pavement drainage, width, grade, and roughness, at locations with fast-evolving hydroplaning risks. To facilitate targeted and precise mitigation plans over road sections with high HpR, future work may focus on reducing uncertainties in HRE estimates, such as by developing a higher resolution elevation data of the pavement surface. Airborne and space-borne LiDAR has the potential to serve such data at continental scale. Follow-up studies may also focus on attribution of the source of heavy rainfall events leading to increased frequency of HREs, and mapping of how they might change in future. These suggestions for novel data applications and analyses are expected to provide a more resolved and accurate HRE estimate, thus facilitating targeted and precise mitigation plans over road sections with high HpR.

## Supporting information

S1 File (DOCX)
