# Supplementary material for: Rainfall-induced hydroplaning risk over road infrastructure of the continental USA
Source: PLoS One. 2022 Aug 31;17(8):e0272993. doi: 10.1371/journal.pone.0272993 (PMC9432704; doi:10.1371/journal.pone.0272993)
Supplement: S1 File — (DOCX) [file pone.0272993.s001.docx]

**Supplementary Information for**

Rainfall-induced hydroplaning risk over road infrastructure of the continental USA

**This PDF file includes:**

Supplementary Figures: Figures S1 to S17

Supplementary Tables: Tables S1 to S2

**Supplementary figures**


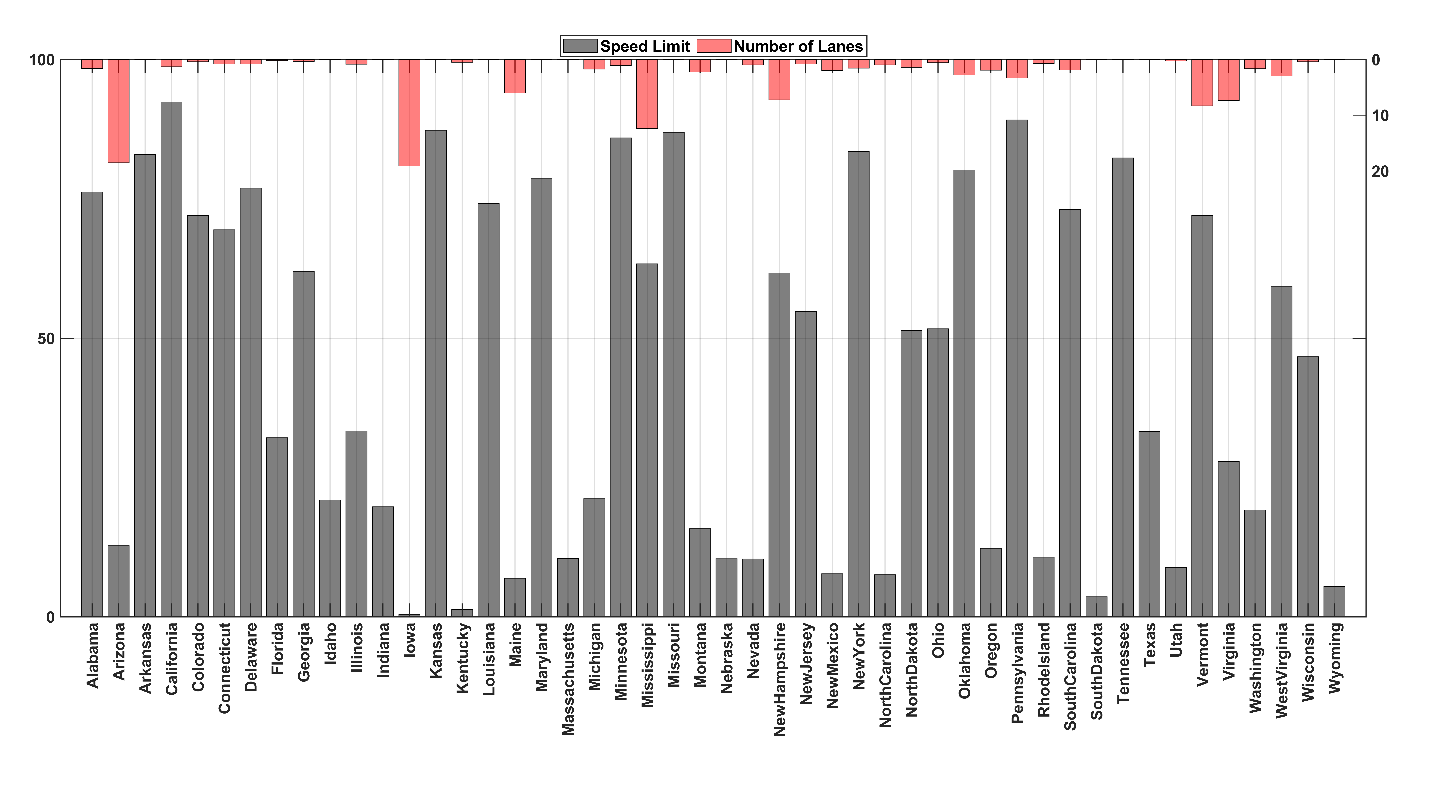


**S1 Fig. State-wise missing data of speed limit (left axis) and number of lanes (right axis, inverted) expressed as fraction (%) of total number of road sections in HPMS road network data within that state.** Data regarding number of lanes are available for most road sections, with maximum missing data being in Iowa (19%). The speed limit data, however, have large gaps with around fifty percent of the states lacking the speed limit data for more than fifty percent of the road section. Modal approach is used for imputation of the missing data for speed limit.


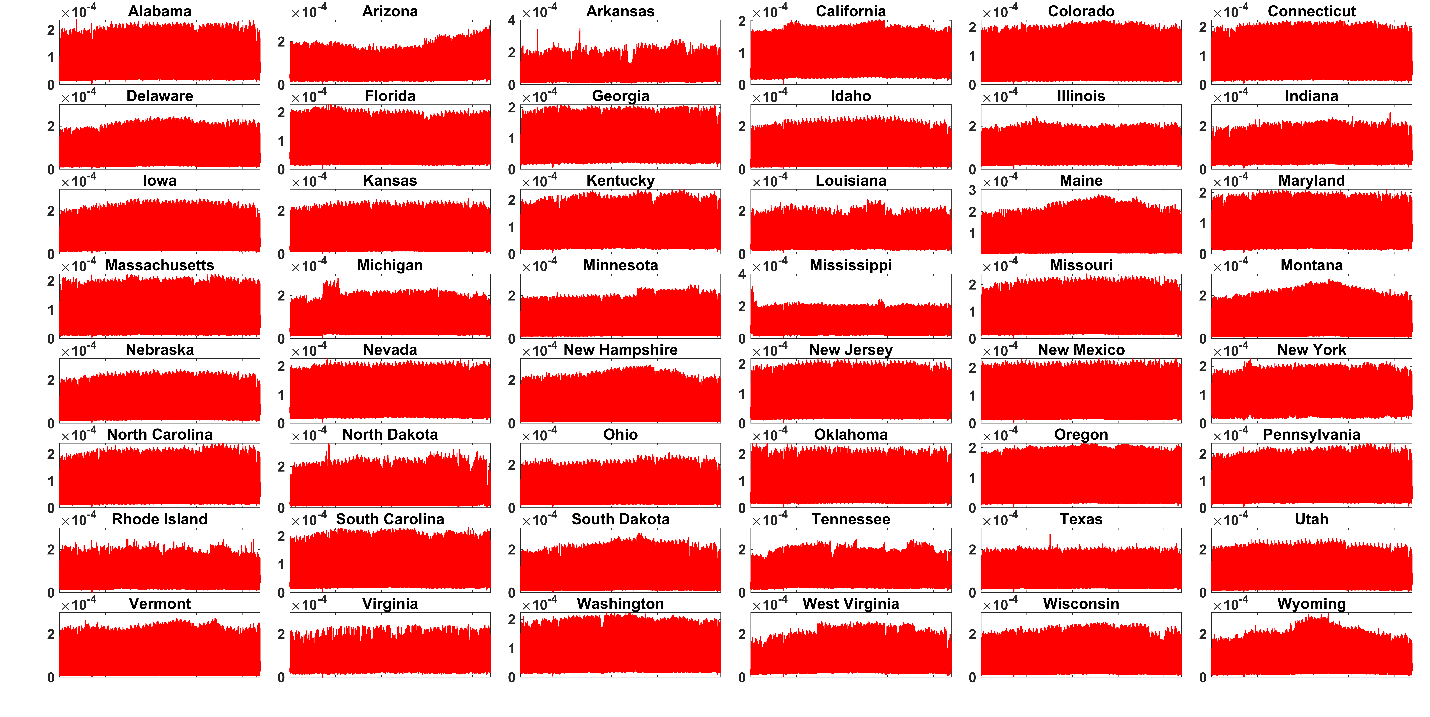


**S2 Fig. State wise traffic volume fractions**. The plot shows the multiyear (2011-2017) average of hourly variation of fraction of total annual traffic volume. The x-axis has 8784 (= 24 x 366) data points.


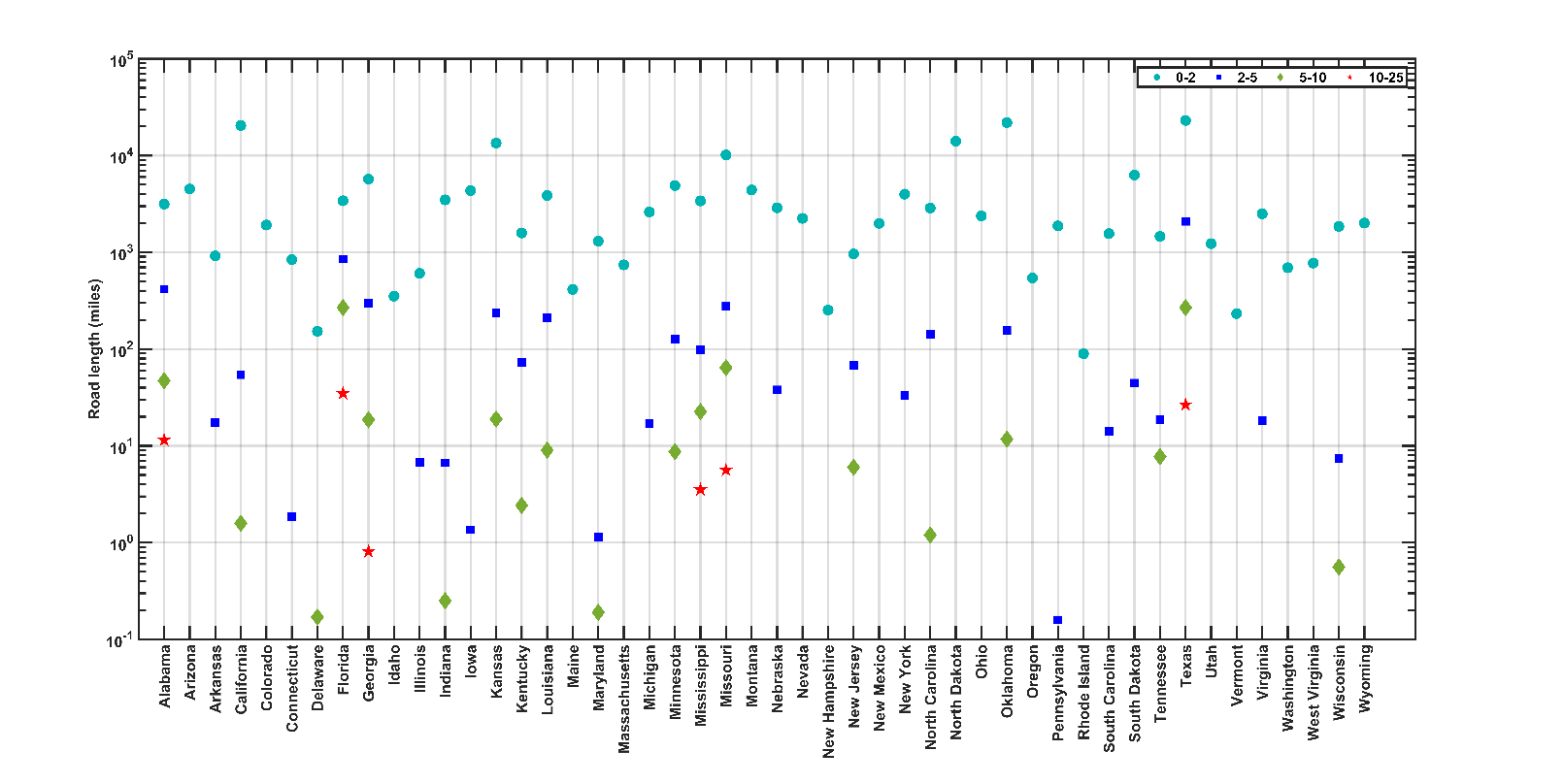


**S3 Fig. Cumulative lengths of road sections experiencing HREs (for MTD = 0.04 inch) in each state of the CONUS.** The southern states of Alabama, Florida, Georgia, Mississippi, Missouri, and Texas consist of road sections with more frequent (> 10 per year) occurrences of HREs implying high HpR.


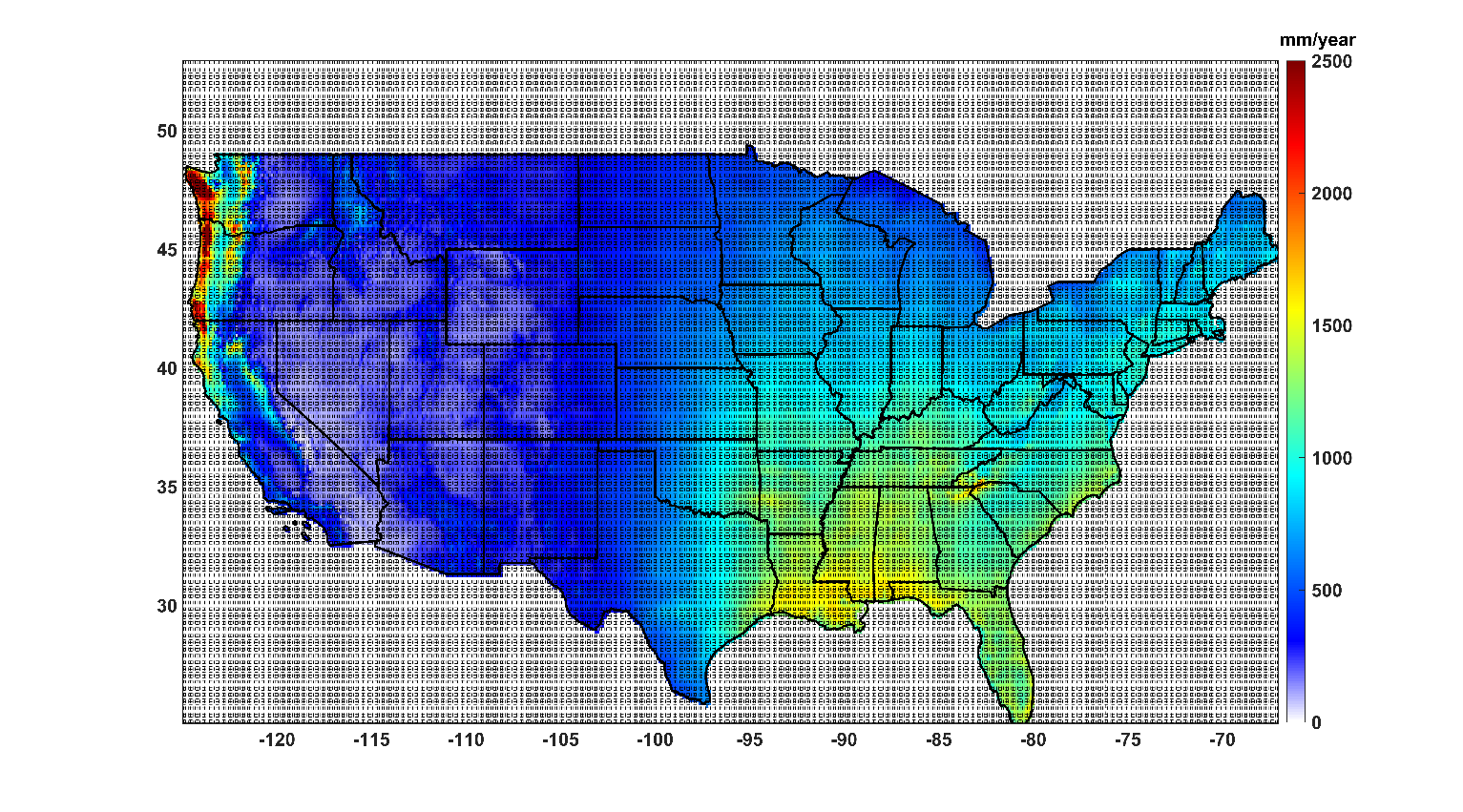


**S4 Fig. Mean annual rainfall over the CONUS during 1980-2017.**


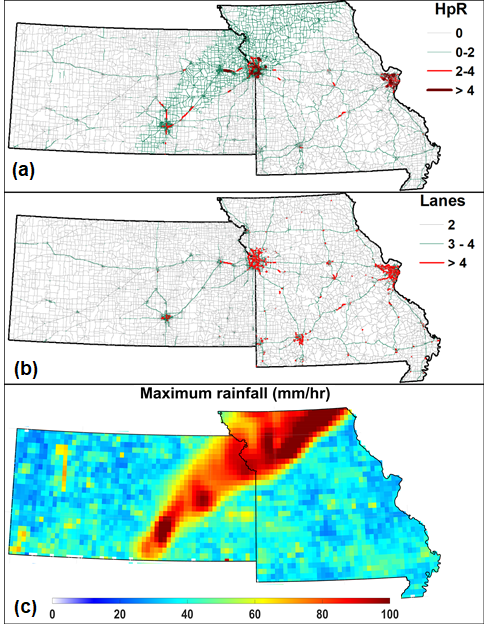


**S5 Fig. Triangular spatial pattern (a) of HREs visible over Kansas and Missouri.** The five inputs that are required for HRE calculations viz. rainfall, mean texture depth (MTD), cross slope, speed limit, and flow path length are analyzed over a part of the study region. Since, the cross slope and MTD are assumed to be identical over entire CONUS for this simulation, the other three factors are to be the reason for this pattern. The number of lanes (which is used to evaluate the flow path length) does not differ significantly between inside and outside of the triangular pattern (b). Within the triangular region, about 70 percent of the road sections have number of lanes equal to two and speed limit equal to 55mph. Based on Gallaway-Huebner equations, it requires at least 66.87mm rainfall in one hour to trigger HREs over a two-lane road with MTD equal to 0.04 inch and cross slope 2 percent. The maximum rainfall that is received over the study region during 1980-2017 shows a spatial pattern matching that of the HREs (c). Notably, inside the triangular region the maximum rainfall magnitude is above 75mm (in one hour) while it is less than 50mm (i.e., also less than 66.87mm) outside of it. This dichotomy leads to the unusual triangular pattern in HREs.


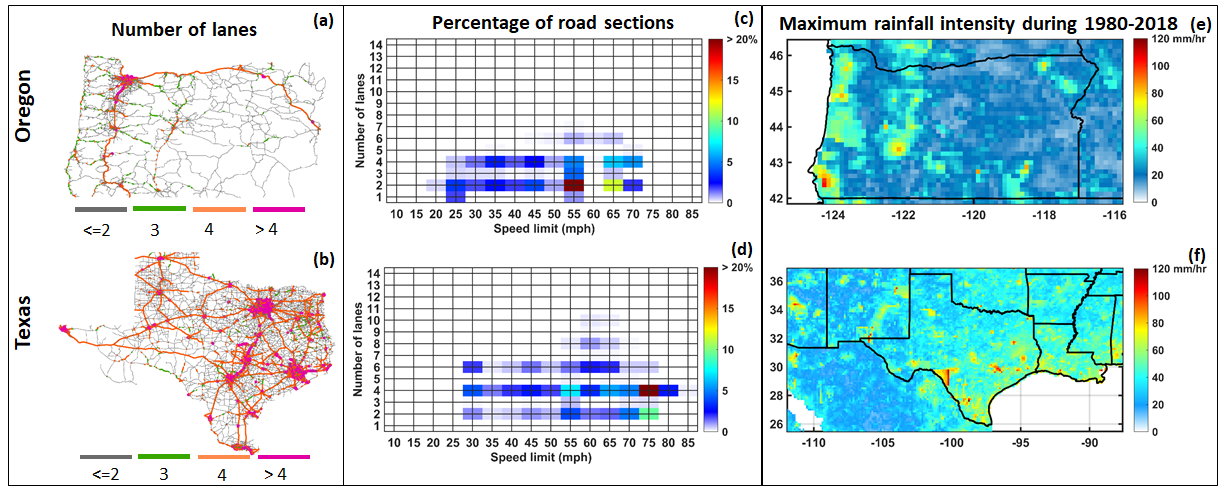


**S6 Fig. Comparison of pavement characteristics and rainfall extremes between Oregon and Texas.** The leftmost panels show the map of number of lanes for road sections in (a) Oregon and (b) Texas, respectively. The center panels show the percentage of road sections in each state with a given speed limit. The rightmost panels show the map of maximum rainfall intensity in (e) Oregon and (f) Texas. A lower annual frequency of HREs in Oregon for most of the road section is attributable to multiple factors: 1) number of lanes: Overall, 69% of the road sections in Oregon have a width less than or equal to two lanes. A smaller number of lanes translates to a smaller overland flow contribution area and hence lower water ponding depth. Comparatively, 43% of the road sections in Texas are two lanes or less; 2) low speed limits: A higher fraction of road sections has lower speed limits in Oregon w.r.t. Texas (Figures S4-c&d). For example, the percentage of road sections with speed limit 45mph (lowest threshold for HP to occur as per Gallaway-Huebner equation) is below is 27% in Oregon as compared to 18% in Texas. Lower vehicle speeds reduce the HpR, and 3) low frequency and sparse occurrence of extreme rainfall that would trigger HREs (Figure S4-e&f) i.e., precipitation intensities that are higher than the threshold intensity needed to result in a HRE at a given road section. For example, in Oregon, only 8% of precipitation grids receive a precipitation event to cause HRE within it. In contrast, the corresponding magnitude in Texas is 86%.


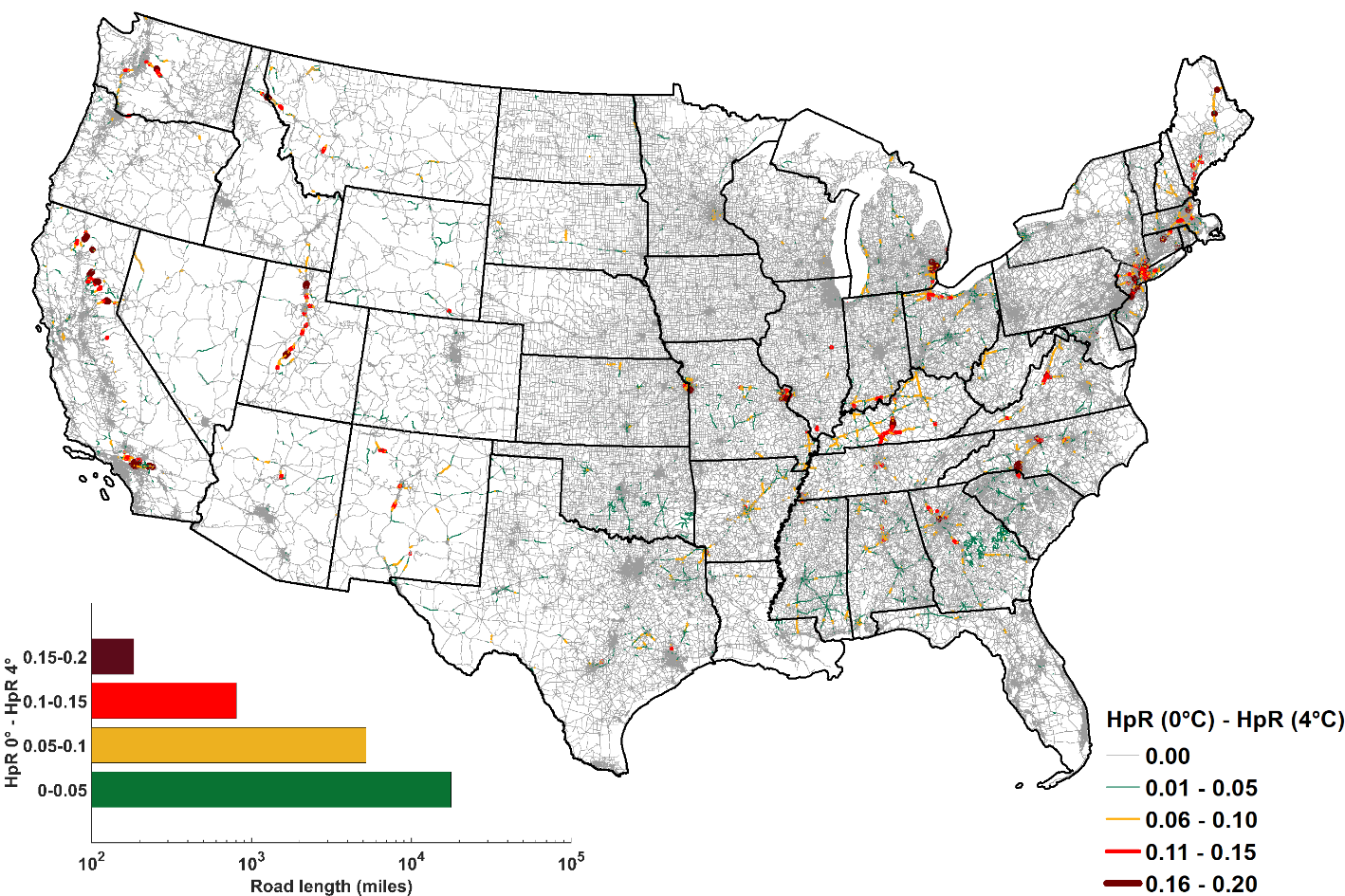


**S7 Fig. Difference in HpR when liquid phase of precipitation is identified based on a temperature threshold of 0°C vs. 4°C.** HpR obtained while considering a 4°C threshold to identify rain is shown in Figure 1. Notably, the maximum difference in HpRs between the two scenarios is only 0.2 and is observed over less than 1 percent of total length of road sections.


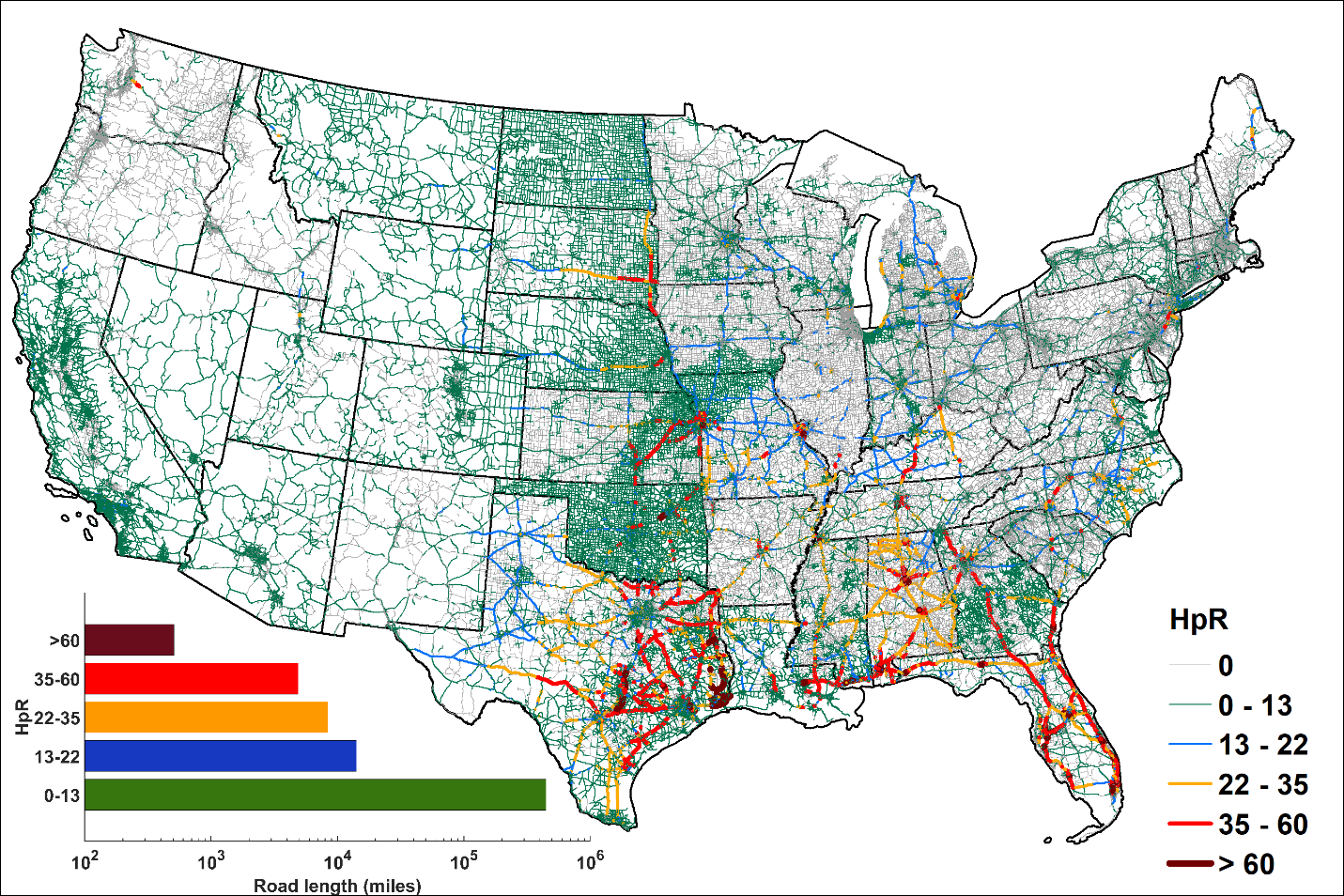


**S8 Fig. HpR, quantified as average annual frequency of HREs, for MTD = 0.004 inch.** A corresponding plot for MTD = 0.04 inch is shown in Figure 1. Overall, 42 % of road sections (greater than 475,287 miles of road length) within the CONUS encounter HpR greater than zero. The frequency of HREs ranges from 0 to 150, with HREs in the range 0-13 affecting 88% of road sections (447,370 miles of road length), 13-22 affecting 6% (14,104 miles) of road sections, 22-35 affecting 3.4% (8,413 miles) of road sections, 35-60 affecting 2% (4,890 miles) of road sections, and 60-150 affecting 0.2% (509 miles) of road sections. The southern part of CONUS encompasses 94% of road sections encountering greater than 60 HREs per year.


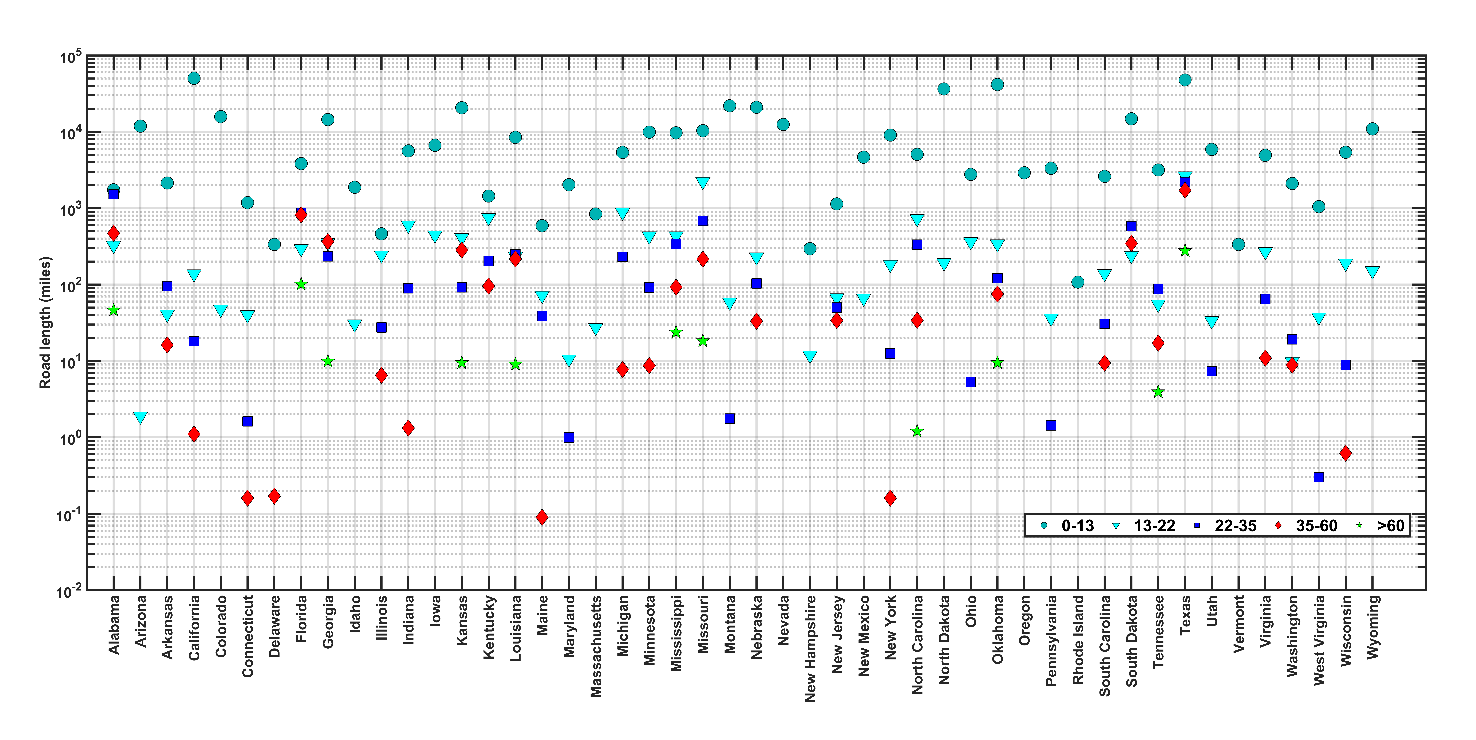


**S9 Fig.** **Cumulative lengths of road sections experiencing HREs for MTD = 0.004 inch in each state of the CONUS.** Seven (of top 10) states viz. Alabama, Florida, Georgia, Louisiana, Oklahoma, Texas, and Mississippi belong to the southern CONUS, where very high average annual HREs are encountered (> 60).


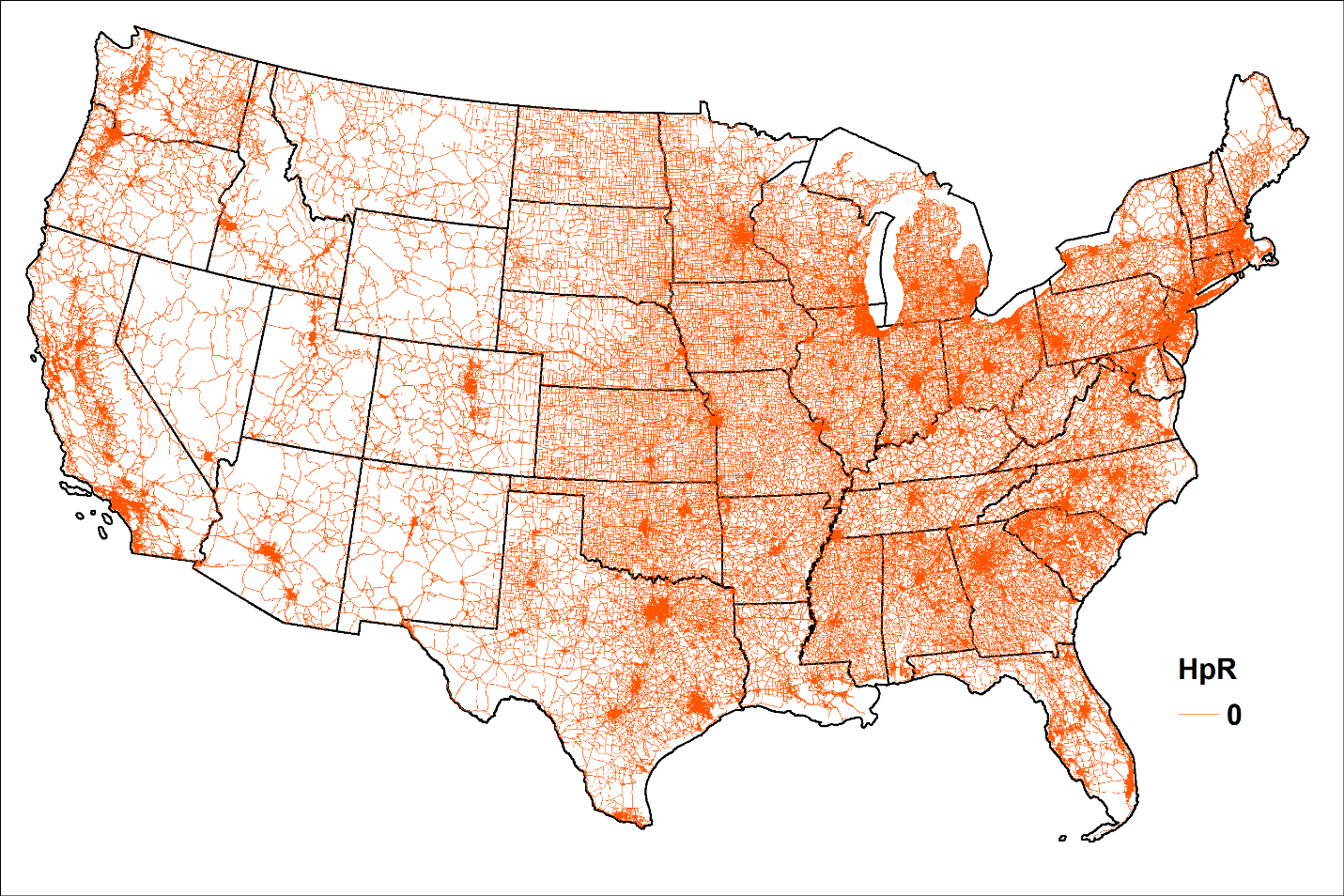


**S10 Fig. same as figure 1, but for MTD = 0.4 inch. With increase in MTD, HREs cease to occur, with HpR dropping to zero.**


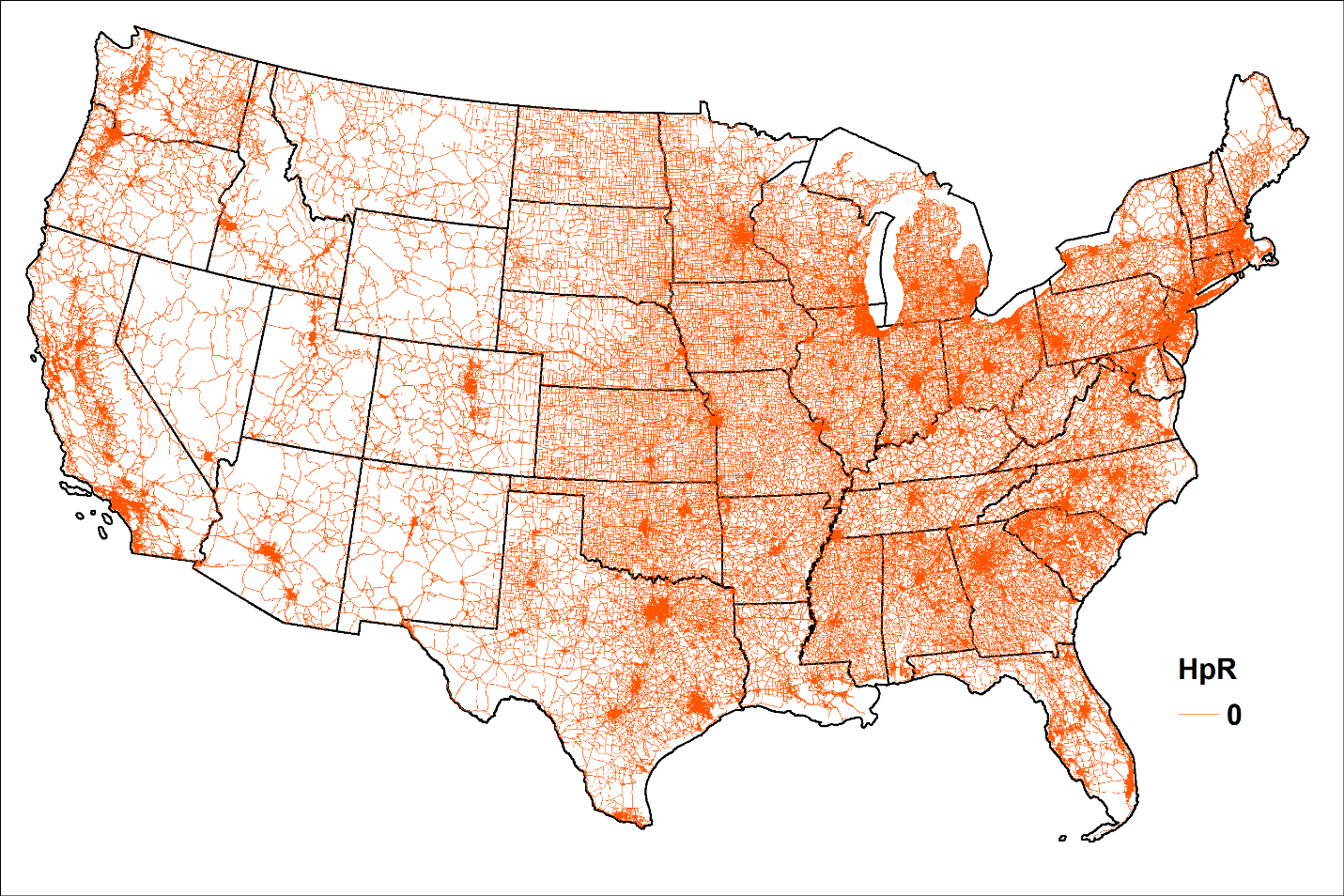


**S11 Fig. same as figure 1, but for MTD = 0.8 inch. With increase in MTD, HREs cease to occur, with HpR dropping to zero.**


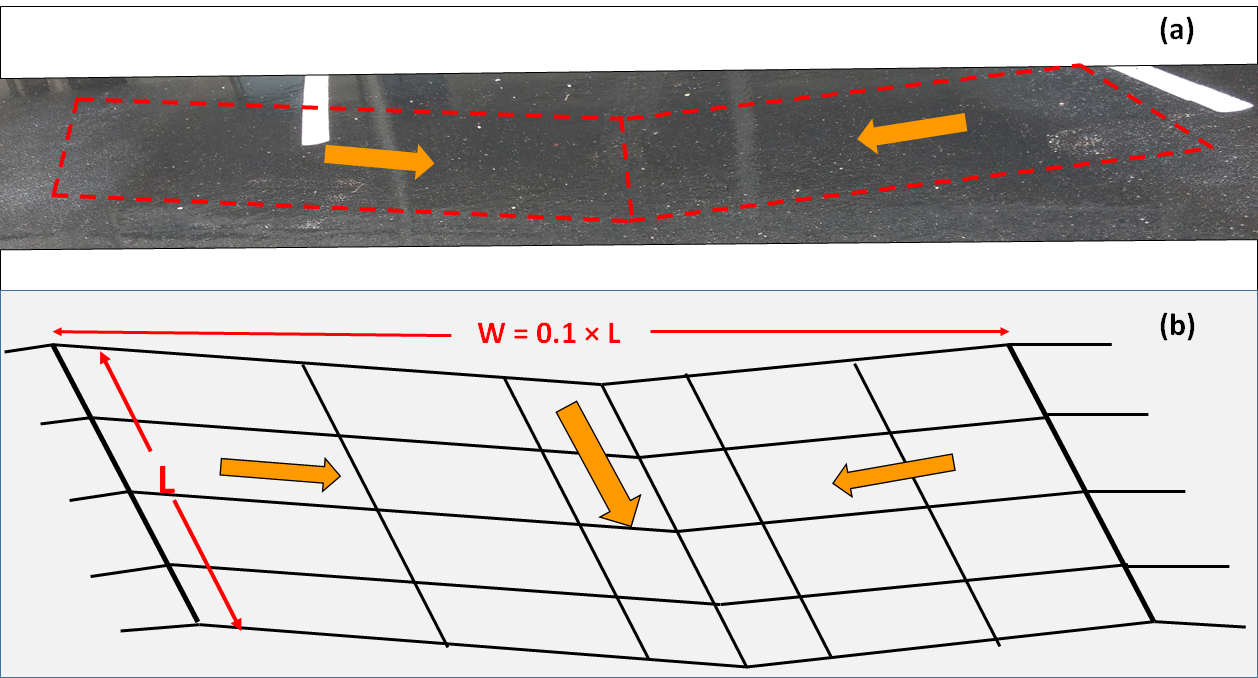


**(a)**

**S12 Fig. Undulations over the pavement cause localized ponding (a). A conceptual diagram of the flow accumulation basin is shown in the lower panel (b).** For an effective drainage width, *W*, of 0.1L (L is the pavement width), effective flow length or the flow contribution area per unit width at the drainage location is 0.1L^2^.

The effective flow length variable implicitly captures the contribution of runoff amount draining through a point on ponded water depth (see Equation 1), and hence the hydroplaning speed (see Equation 2). Under ideal conditions, i.e., for a smooth pavement free of any local depressions, lateral drainage of ponded water is mainly driven by the cross slope. In this case, the width of the pavement represents the flow path length. In real conditions, a pavement usually has local depressions, as conceptualized by a v-shaped catchment (shown in Fig S12 (a)). Under these circumstances, the runoff through the drainage point is contributed from the entire catchment rather than along a linear element only. The effective flow length, and hence the ponded water depth, in this situation will generally be relatively larger.
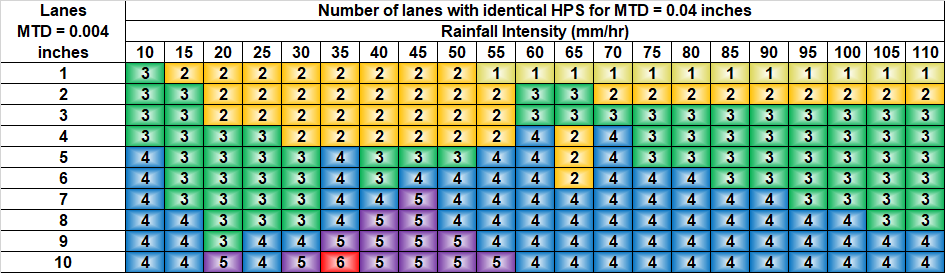


**S13 Fig. Number of lanes (listed in each square) of pavement with MTD = 0.04 and a modified effective flow path that will produce the same HPS (and hence HpR) as that with MTD = 0.004 inch and flow path length equal to width of the road.** The modified flow path is evaluated assuming a flow contribution area of 0.1L^2^ due to local undulations (as depicted in Figure S7). The evaluation is performed for a range of rainfall intensities.


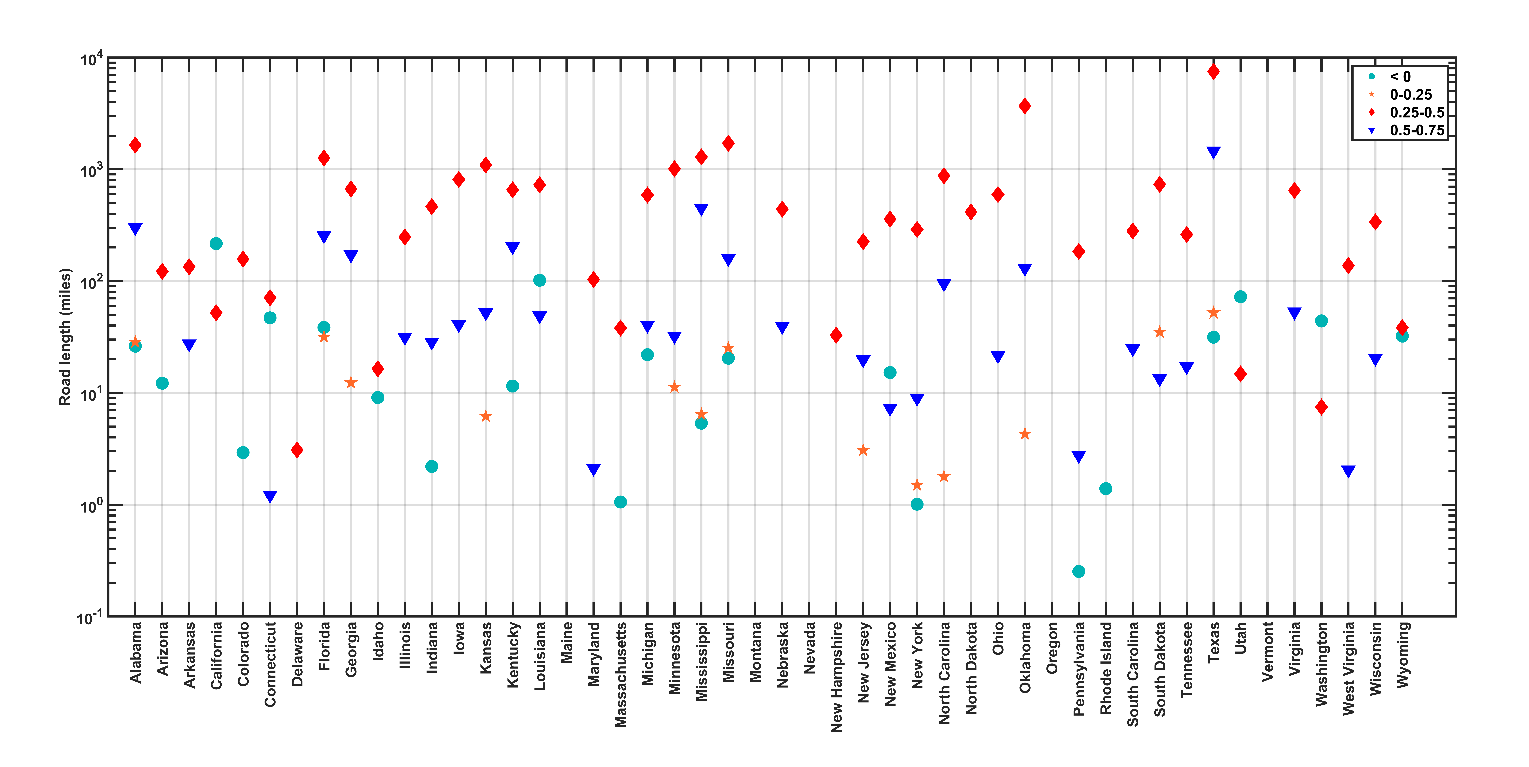


**S14 Fig. Cumulative lengths of road sections experiencing statistically significant trend in annual frequency of HREs.** The evaluation is performed for MTD = 0.04 inch. Over 33,887 miles of road sections (~5.3 % of overall road section within the CONUS) show temporally increasing trend. Six southern CONUS states (Texas, Oklahoma, Alabama, Mississippi, Florida, and North Carolina) are on the list of top ten states with maximum road length experiencing HREs with positive trends. 714 miles of road sections (0.12% of roads experiencing HREs) exhibit a negative trend in HREs. The evaluation is performed for MTD = 0.04 inches.


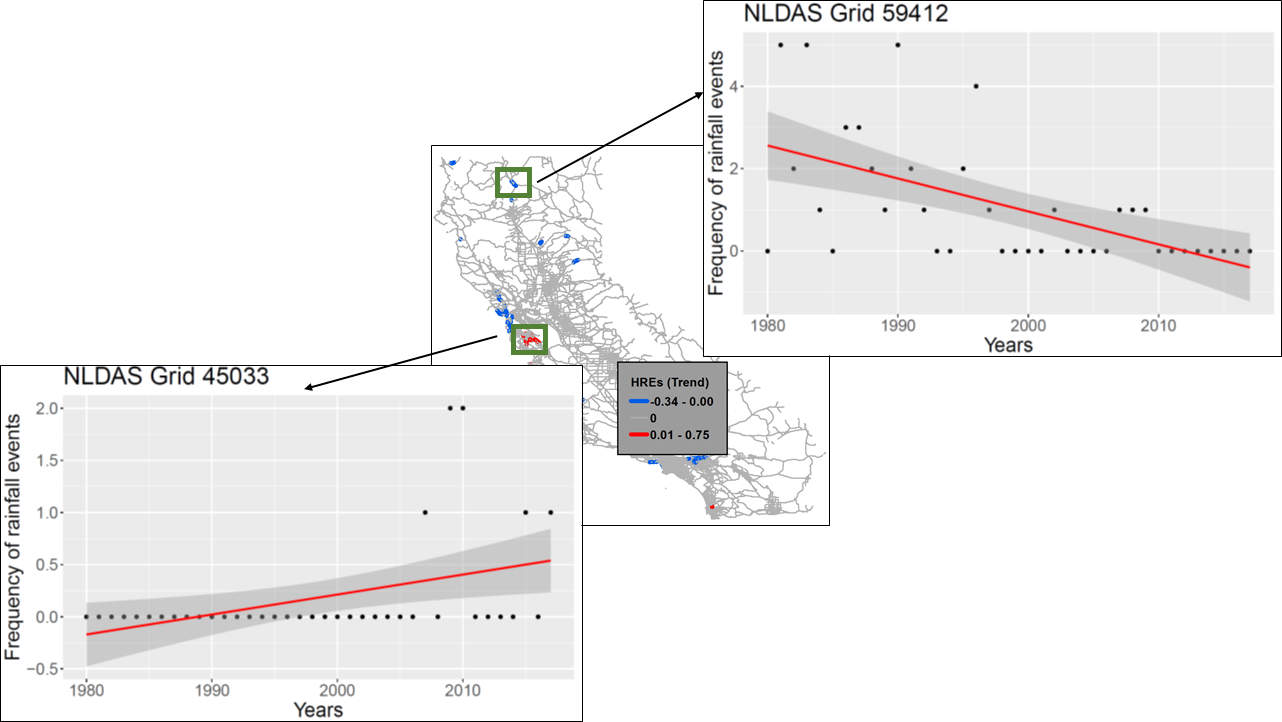


**S15 Fig. Root cause analysis of temporally evolving HpR with negative trend of HREs.** Rainfall time series at two NLDAS grids (number 59,412, Lon: -122.4375, Lat: 41.0625 and 45,033, Lon: -121.8125, Lat: 37.1875), located within California are analyzed. As an example, a road section with six lanes and a speed limit of 65mph is considered. As per Gallaway-Huebner equation (Equations 1 and 2), the rainfall intensity required to generate HREs over the road sections with six lanes is 17.3mm/hr. Annual frequency of rainfall events with intensity greater than 17.3mm/hr is obtained and trend analysis is carried out. Trend (red line) of frequency of rainfall above the threshold intensity (shown in black dots) confirms that decrease in rainfall extremes above the threshold is the root cause behind negative HRE in NLDAS grid 59,412. Similar analysis over grid 45,033 indicates a positive trend in annual frequency of events above the threshold.


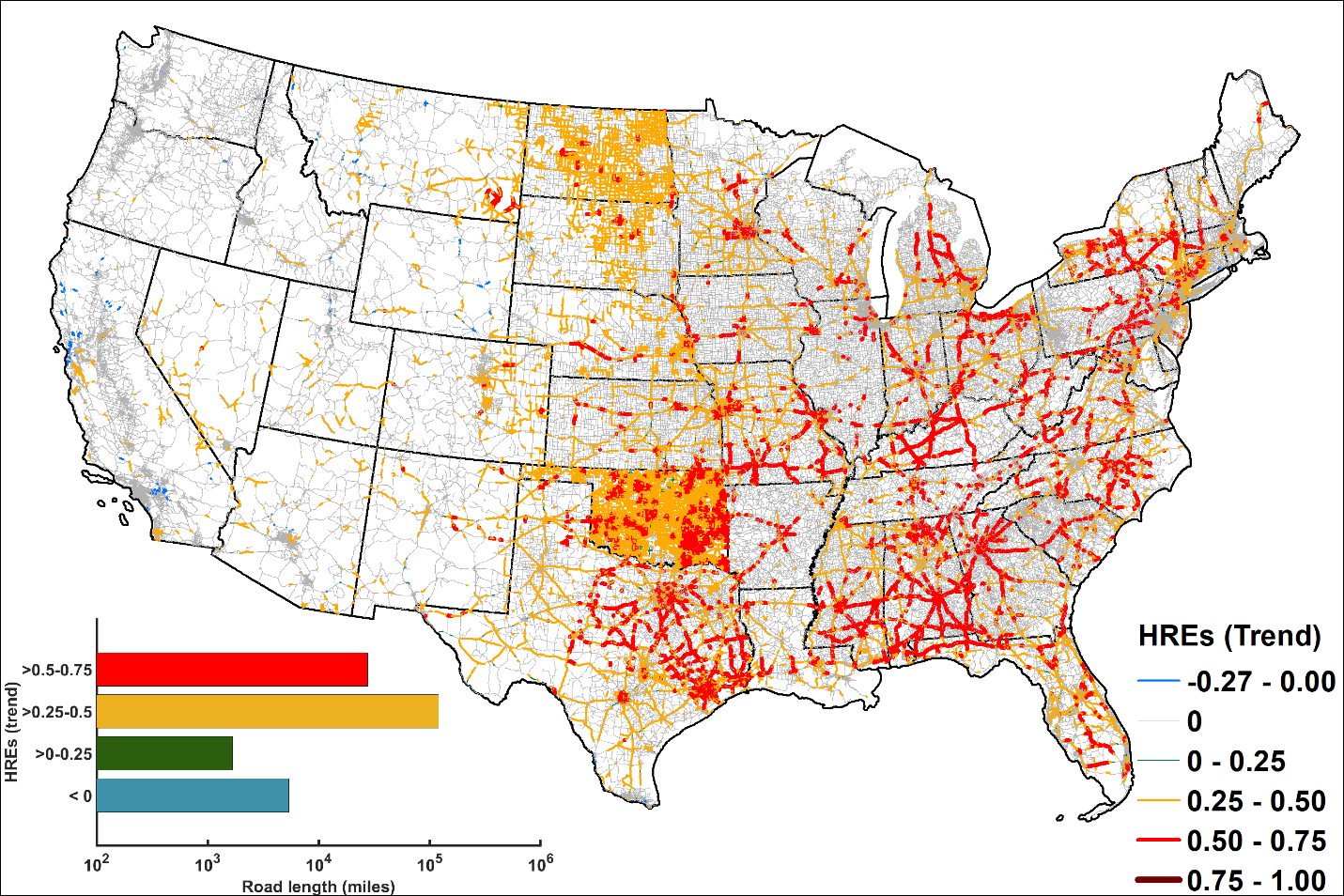


***SNS: Statistically not significant**

**OR *SNS**

**S16 Fig. Temporal trends in hydroplaning risk (HpR) during 1980-2017 for MTD = 0.004 inch.** With reduction in MTD from 0.04 to 0.004, a larger fraction of roads (16% vs. 5.3%) experience statistically significant trends. Notably, this dominance is still limited to the eastern half of CONUS.


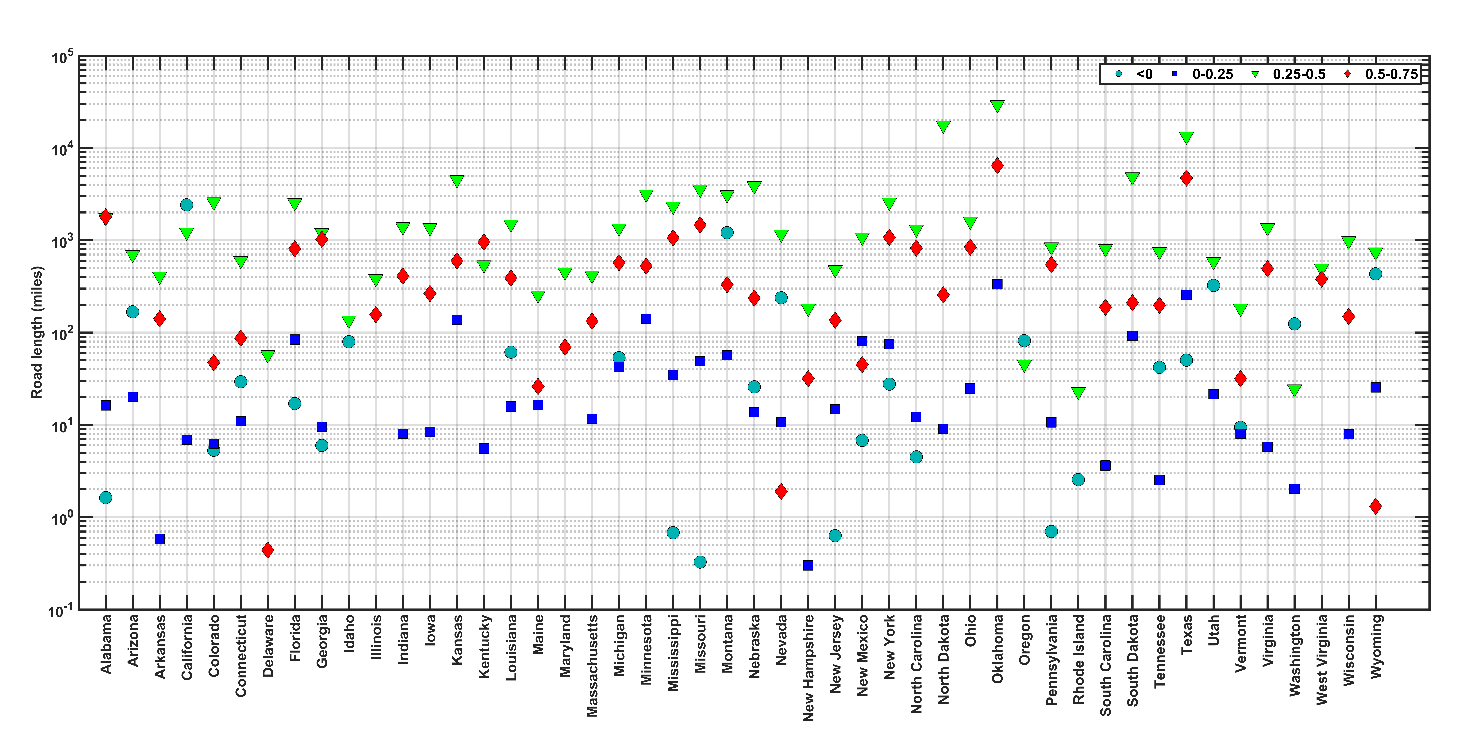


**S17 Fig. Cumulative lengths of road sections experiencing statistically significant trend (p < 0.05) in annual frequency of HREs for MTD = 0.004.** Among the top ten states in terms of lengths of roads showing positive trends in HREs, 6 states (Oklahoma, Texas, Alabama, Missouri, Kansas, Minnesota) also feature in the top ten for MTD = 0.04.

**Supplementary Tables**

**S1 Table.** Ranked list of state-wise length-weighted average of annual HREs (with road section length as weights) and total number of HREs per year for MTD = 0.04 inch. States below 37 degrees latitude and extending westward from the east coast until Texas are considered within the southern United States, and are identified with a superscript “*” below.

| **No.** | **States** | **HREs (length weighted)** | **State** | **Total HREs/year** |  | **No.** | **States** | **HREs (length weighted)** | **State** | **Total HREs/year** |
| --- | --- | --- | --- | --- | --- | --- | --- | --- | --- | --- |
| 1 | Florida^*^ | 0.18 | Texas^*^ | 185235.03 |  | 25 | Maine | 0.015 | North Dakota | 3746.89 |
| 2 | Texas^*^ | 0.134 | Florida^*^ | 79008 |  | 26 | Tennessee^*^ | 0.014 | Illinois | 3294.53 |
| 3 | Missouri | 0.125 | Alabama^*^ | 48823.71 |  | 27 | Maryland | 0.013 | South Carolina^*^ | 3165.34 |
| 4 | Louisiana^*^ | 0.11 | Missouri | 47397.11 |  | 28 | Massachusetts | 0.012 | Connecticut | 2995.5 |
| 5 | Alabama^*^ | 0.099 | California | 36393.26 |  | 29 | Wyoming | 0.011 | Arkansas^*^ | 2981.58 |
| 6 | Oklahoma^*^ | 0.077 | Kansas | 32919.21 |  | 30 | Wisconsin | 0.01 | New Mexico | 2967.11 |
| 7 | Mississippi^*^ | 0.061 | Mississippi^*^ | 25712.13 |  | 31 | Nevada | 0.009 | Arizona | 2711.82 |
| 8 | Kansas | 0.06 | Oklahoma^*^ | 25045.21 |  | 32 | New Hampshire | 0.009 | Massachusetts | 2130.74 |
| 9 | South Dakota | 0.056 | Georgia^*^ | 22969.18 |  | 33 | Arkansas^*^ | 0.009 | Pennsylvania | 2018.84 |
| 10 | Georgia^*^ | 0.05 | Minnesota | 20282.63 |  | 34 | New Mexico | 0.008 | Maryland | 1539.45 |
| 11 | Kentucky | 0.041 | Louisiana^*^ | 19516.63 |  | 35 | South Carolina^*^ | 0.008 | Wyoming | 1479.08 |
| 12 | California | 0.04 | South Dakota | 16364.71 |  | 36 | Montana | 0.008 | Colorado | 1394.58 |
| 13 | North Carolina^*^ | 0.035 | North Carolina^*^ | 15981.34 |  | 37 | Delaware | 0.008 | Maine | 1258.79 |
| 14 | New Jersey | 0.03 | Michigan | 15328.45 |  | 38 | Ohio | 0.007 | West Virginia | 1157.82 |
| 15 | New York | 0.025 | New York | 13443.53 |  | 39 | West Virginia | 0.007 | Montana | 1029 |
| 16 | Virginia | 0.025 | Kentucky | 10345.97 |  | 40 | Rhode Island | 0.006 | Utah | 973.39 |
| 17 | Minnesota | 0.025 | Iowa | 9026.76 |  | 41 | Illinois | 0.006 | Washington | 679.84 |
| 18 | Nebraska | 0.023 | Ohio | 8472.18 |  | 42 | Pennsylvania | 0.005 | New Hampshire | 465.37 |
| 19 | Indiana | 0.022 | New Jersey | 8113 |  | 43 | Colorado | 0.004 | Nevada | 265.58 |
| 20 | Iowa | 0.021 | Virginia | 7778.16 |  | 44 | Utah | 0.004 | Delaware | 247.53 |
| 21 | North Dakota | 0.019 | Indiana | 7751.63 |  | 45 | Vermont | 0.003 | Idaho | 199.29 |
| 22 | Connecticut | 0.018 | Nebraska | 6901.24 |  | 46 | Washington | 0.002 | Rhode Island | 170.84 |
| 23 | Michigan | 0.017 | Wisconsin | 5552.66 |  | 47 | Idaho | 0.001 | Oregon | 148.68 |
| 24 | Arizona | 0.016 | Tennessee^*^ | 4047.63 |  | 48 | Oregon | 0.001 | Vermont | 123.26 |

**S2 Table.** Similar to table S1, but for MTD = 0.004 inch.

| **No.** | **States** | **HREs (length weighted)** | **State** | **Total HREs/year** |  | **No.** | **States** | **HREs (length weighted)** | **State** | **Total HREs/year** |
| --- | --- | --- | --- | --- | --- | --- | --- | --- | --- | --- |
| 1 | Missouri | 2.479 | Texas^*^ | 2915881.5 |  | 25 | Connecticut | 0.429 | New Jersey | 95427.89 |
| 2 | Florida^*^ | 2.184 | Alabama^*^ | 992885.11 |  | 26 | Iowa | 0.385 | New Mexico | 95017.18 |
| 3 | Texas^*^ | 2.149 | Florida^*^ | 929411.76 |  | 27 | West Virginia | 0.374 | North Dakota | 87989.03 |
| 4 | Oklahoma^*^ | 2.134 | Missouri | 924266.63 |  | 28 | New Jersey | 0.362 | Pennsylvania | 85938.39 |
| 5 | Alabama^*^ | 2.08 | Kansas | 530789.68 |  | 29 | New Hampshire | 0.288 | Arizona | 84967.61 |
| 6 | South Dakota | 1.642 | California | 506447.58 |  | 30 | Massachusetts | 0.27 | Illinois | 79221.32 |
| 7 | Louisiana^*^ | 1.5 | Mississippi^*^ | 474914.47 |  | 31 | New Mexico | 0.267 | Tennessee^*^ | 74788.95 |
| 8 | Kentucky | 1.21 | South Dakota | 456114.39 |  | 32 | Tennessee^*^ | 0.266 | Connecticut | 69276.66 |
| 9 | Mississippi^*^ | 1.129 | Michigan | 453095.76 |  | 33 | Nevada | 0.248 | Utah | 67450.97 |
| 10 | Kansas | 0.95 | Oklahoma^*^ | 432150.84 |  | 34 | Utah | 0.24 | West Virginia | 62437.24 |
| 11 | Georgia^*^ | 0.865 | Georgia^*^ | 401061.32 |  | 35 | Pennsylvania | 0.221 | Colorado | 60152.55 |
| 12 | Wyoming | 0.827 | Minnesota | 371958.29 |  | 36 | Wisconsin | 0.216 | South Carolina^*^ | 55859.18 |
| 13 | North Carolina^*^ | 0.71 | North Carolina^*^ | 320409.82 |  | 37 | Ohio | 0.211 | Arkansas^*^ | 55679.84 |
| 14 | Montana | 0.608 | Kentucky | 300389.89 |  | 38 | Colorado | 0.191 | Maine | 48400.13 |
| 15 | Indiana | 0.596 | Louisiana^*^ | 268108.79 |  | 39 | Idaho | 0.164 | Massachusetts | 46859.13 |
| 16 | North Dakota | 0.583 | New York | 265154.97 |  | 40 | Arkansas | 0.163 | Idaho | 31802.29 |
| 17 | Maine | 0.575 | Ohio | 261419.42 |  | 41 | Maryland | 0.157 | Washington | 25310.89 |
| 18 | California | 0.568 | Indiana | 220610.58 |  | 42 | South Carolina^*^ | 0.151 | Maryland | 18599.84 |
| 19 | Virginia | 0.545 | Iowa | 171784.71 |  | 43 | Rhode Island | 0.137 | Nevada | 16665.82 |
| 20 | Michigan | 0.527 | Virginia | 166773.32 |  | 44 | Illinois | 0.137 | New Hampshire | 15483.05 |
| 21 | New York | 0.517 | Nebraska | 144906.89 |  | 45 | Vermont | 0.135 | Vermont | 6668.76 |
| 22 | Nebraska | 0.515 | Wisconsin | 112503.32 |  | 46 | Delaware | 0.087 | Oregon | 4475.47 |
| 23 | Minnesota | 0.472 | Wyoming | 112384.89 |  | 47 | Washington | 0.075 | Rhode Island | 3671.32 |
| 24 | Arizona^*^ | 0.437 | Montana | 100268.92 |  | 48 | Oregon | 0.014 | Delaware | 2752.26 |
